# Nitrogen Fixation by MoSn_3_ and MoSn_5_ Clusters

**DOI:** 10.1021/acsomega.6c02579

**Published:** 2026-07-24

**Authors:** Jesús Iván Salazar-Barrientos, Thamara V. Salazar-Barrientos, Peter T. Cummings, José Manuel Guevara-Vela, Tomás Rocha-Rinza

**Affiliations:** † Instituto de Química, Universidad Nacional Autónoma de México, Circuito Exterior, Ciudad Universitaria s/n, Ciudad de México CP 04510, Mexico; ‡ Canadian Centre for Research in Advanced Fluorine Technologies (C-CRAFT) and Department of Chemistry and Biochemistry, 4512 University of Lethbridge, Lethbridge, Alberta T1K 3M4, Canada; § School of Engineering and Physical Sciences, 3120 Heriot-Watt University, Edinburgh EH14 4AS, Great Britain

## Abstract

The Haber–Bosch process (HBP) for the conversion of nitrogen into ammonia is arguably one of the most important chemical reactions in human history because it is the basis for the large-scale production of fertilizers and hence of global food production. Nevertheless, the HBP contributes in a decisive manner to the production of CO_2_ and other global warming gases, since it is an extremely energy-intensive process. Consequently, the development of alternative conditions to reduce its negative environmental impact is of the highest priority. In this study, we investigate the detailed mechanism of nitrogen fixation by molybdenum-doped tin clusters MoSn_3_ and MoSn_5_, building upon previous work that demonstrated the potential of these complexes for activating the nitrogen molecule (*Inorg. Chim. Acta* **2025,** *577,* 122493). Using a combination of density functional theory and wave function analyses, we explored the stepwise reduction of N_2_ into NH_3_ on the surface of the above-mentioned clusters. Our results reveal the role of molybdenum as an excellent catalytic center for the HBP and the properties of the surrounding tin atoms as scaffolds for hydrogen adsorption. We identified the intermediates and transition states along six different reaction pathways, providing insights into the kinetic aspects of the nitrogen fixation process using MoSn_3_ and MoSn_5_ as catalysts. We also demonstrated that (i) the ability of these clusters to form hydrides mimics the biological activity of the nitrogenase enzyme and (ii) how the strong triple bond in dinitrogen, N≡N, is successively weakened in key steps of the reaction pathways. Overall, this work deepens our understanding of nitrogen fixation via heterogeneous catalysis, and it provides valuable insights for the rational design of more efficient and sustainable catalysts for ammonia synthesis.

## Graphical abstract

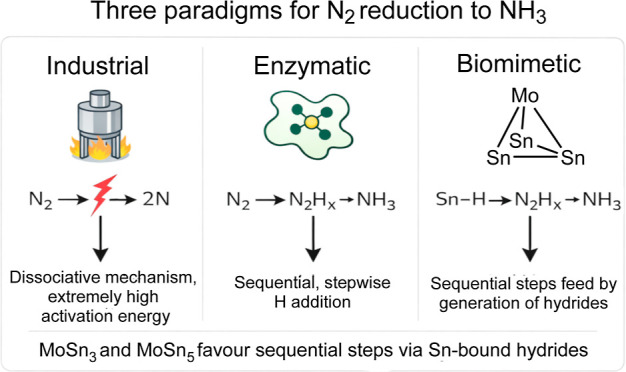

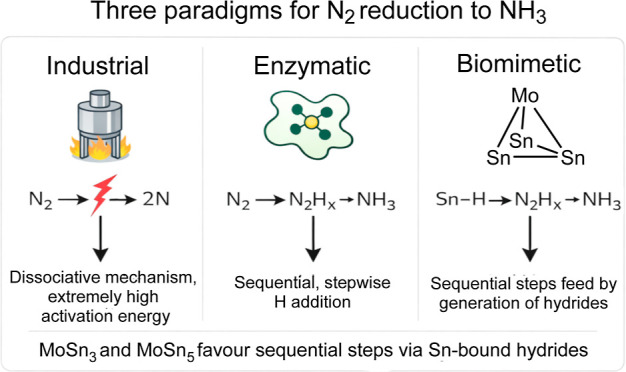

## Introduction

Approximately 78% of Earth’s atmosphere is composed of molecular nitrogen, which is chemically and biologically inert in its natural (gaseous) form. Because of the inertness of the N_2_ molecule, the production of ammonia (NH_3_) from nitrogen and hydrogen, known as the Haber–Bosch process (HBP), is one of the most important chemical reactions in human history.^1^ The HBP has revolutionized agriculture by enabling the large-scale production of synthetic fertilizers, which in turn has supported an unprecedented increase in global food production.^2^ It is estimated that nearly half of the current global population is sustained thanks to the ammonia produced via the HBP.^1^ Moreover, approximately 50% of the nitrogen found in human tissues comes from this process.^2^ However, the HBP is highly energy-intensive, and it requires extreme pressures (150–300 atm) and temperatures (700–800 K) to achieve the desired conversion of N_2_ and H_2_ into NH_3_.^3,4^ This demand of energy is largely met by the burning of fossil fuels, making the process a significant contributor to global carbon emissions.^5^ Hence, there is a pressing need to develop more sustainable and energy-efficient methods for nitrogen fixation and the production of ammonia.

In this regard, the industrial synthesis of NH_3_ from N_2_ proceeds via a dissociative mechanism, in which H or H_2_ reacts with Fe-adsorbed nitrogen species.^6–8^ The industrial HBP requires the splitting of the N≡N bond in the early stages of the reaction, as shown in the left panel of Figure 1. The breaking of this chemical bond is a quite energetically demanding process.^9–11^ In contrast, the fixation of nitrogen in nature occurs via a sequential mechanism (right panel of Figure 1) under much milder conditions than those required for the industrial HBP. The overall reaction occurs through a series of redox reactions catalyzed by nitrogenase enzymes^12,13^ containing metals such as Fe, Mo, and Cu in their active site. In this mechanism, H or H_2_ mainly reacts with Mo-adsorbed N_2_ species, hydrogenating the N_2_ and weakening the N≡N bond.

**Figure 1:**
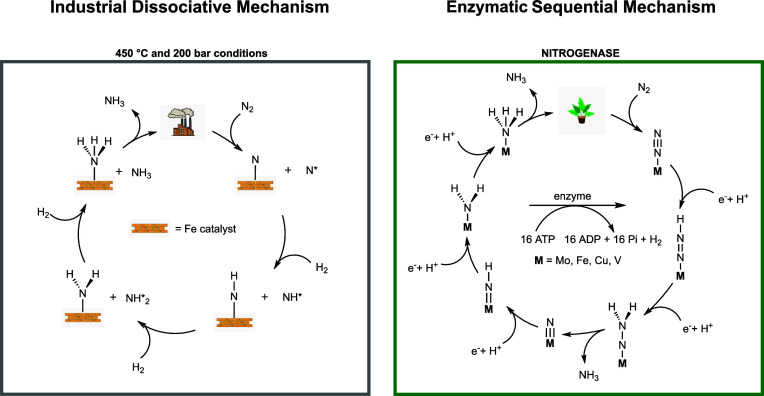
Industrial^7^ and enzymatic^14^ mechanisms for N_2_ fixation.

There have been efforts focused on developing catalytic processes for ammonia production that are more efficient and more environmentally friendly than the industrial HBP.^15^ These efforts include milder reaction conditions and alternative hydrogen sources that do not rely on natural gas or coal. Schrock and Yandulov reported experimental evidence for the fixation of N_2_ via associative mechanisms involving a molybdenum center as a catalyst.^16^ More recently, Batov et al. identified a uranium triamidoamine complex capable of reducing N_2_ to NH_3_ via a sequential reduction.^17^ Additionally, Li et al. reported the formation of ammonia via electrosynthesis on ruthenium nanoclusters.^18^ From a theoretical perspective, Zeinalipour-Yazdi conducted a comparative analysis of various Fe and Ru catalytic mechanisms for ammonia synthesis on different industrial catalysts, metal nitrides, and nanocluster-supported systems using density functional theory (DFT).^19^ In another study, the same group reported both associative and dissociative mechanisms for ammonia production using a Co_3_Mo_3_N catalyst in the presence of surface defects, suggesting that ammonia synthesis can be enhanced under milder conditions.^20^ Additionally, single-atom catalysts (SAC)^21,22^ and single-cluster catalysts^23^ have been considered for the production of ammonia. For example, Liu et al. studied ammonia production on a Fe_3_ cluster anchored on the θ-Al_2_O_3_(010) surface, while Zhang and co-workers developed a nanosecond pulsed spark discharge for sustainable nitrogen fixation at ambient conditions sustained by free-radical-chain reactions.^24^ Finally, in a previous study by our group,^25^ we investigated the stability of MoSn_*n*_ clusters and their ability to activate the N_2_ molecule. The adsorption of N_2_ on the Mo center of the MoSn_3_ and MoSn_5_ clusters induces an elongation of the N≡N triple bond. This bond weakening indicates strong π backbonding from the Mo atom to the antibonding orbital of the N_2_ molecule, which enhances the reactivity of the last-mentioned species. Most importantly, the weakening of the N≡N bond constitutes an essential step in nitrogen activation in the HBP. Interestingly, our analysis revealed that the Mo atom in these clusters carries a negative charge. This feature not only results in a repulsive charge–quadrupole interaction between the Mo center and the N_2_ molecule as discussed below, but it also reduces the capability of the Mo atom to accept the nitrogen lone pair. Therefore, we hypothesize that the negative charge of molybdenum would lead to a diminution of the formation energy of the resulting Mo–N adducts. This finding might be significant, as one of the key challenges in using Mo for N_2_ fixation lies in the formation of too strong Mo–N bonds, a circumstance which can hinder the subsequent bond dissociation step required for ammonia production.^26^

By virtue of these features of Mo and Sn clusters, we elucidated the mechanism for the fixation of N_2_ catalyzed by MoSn_3_ and MoSn_5_ clusters. For this purpose, we employed a combination of (i) DFT calculations and (ii) wave function analyses based on the quantum theory of atoms in molecules (QTAIM) to examine the stepwise reduction of N_2_ to NH_3_. Specifically, we examined the reaction pathways, key intermediates, and transition states associated with different associative mechanisms, aiming to identify the most favorable route for ammonia production in the presence of H atoms. In comparison with metal nitrides and SACs, the MoSn_3_ and MoSn_5_ clusters studied herein exhibit a distinct coordination environment, charge state, and reaction pathways. Whereas nitrides provide active sites embedded in extended lattices and SACs rely on support-defined local coordination,^20–22^ MoSn_3_ and MoSn_5_ contain a discrete Mo center within a finite tin cluster. In addition, the Mo atom in these systems carries a negative charge, unlike the more conventional supported or framework-bound active centers discussed above, which alters the binding and activation of N_2_. Consequently, the reaction pathways are also expected to differ. Rather than following the pathway organization imposed by a rigid lattice or a supported single site, associative nitrogen reduction on MoSn_*n*_ proceeds through a mechanism in which N_2_ activation occurs at Mo while hydrogen accommodation takes place on the surrounding Sn atoms. This distinctive combination makes MoSn_*n*_ a mechanistically different platform for nitrogen fixation relative to nitrides and SACs. Altogether, we provide a deep understanding of the catalytic potential of MoSn_3_ and MoSn_5_ clusters for the fixation of N_2_, and we offer valuable insights into the design of more efficient catalysts for sustainable ammonia production.

## Results and Discussion

### N_2_ Adsorption on MoSn_*n*_ Clusters

As mentioned in the Introduction, we carried out the mechanistic study of N_2_ fixation using the MoSn_3_ and MoSn_5_ clusters as catalysts, motivated by the results previously reported by our group,^25^ which indicated that these systems are particularly stable within the series MoSn_*n*_ (*n* = 2–15). Moreover, these clusters also exhibit in its interaction with N_2_ strong π backbonding from the Mo atom to the antibonding orbital of N_2_, resulting in the activation of the last-mentioned molecule.^25^ The most stable configurations of N_2_ adsorbed on the MoSn_3_ and MoSn_5_ clusters are shown in Figure 2(a). Configurations (A) and (C) in Figure 2(a) present three-center, two-electron interactions in the adsorption of the N_2_ molecules with the Mo center. We identified previously the structures (B) and (D) in Figure 2(a) of the molecular clusters MoSn_3_···N_2_ and MoSn_3_···N_2_.^25^ These structures are stable despite a repulsive charge–quadrupole interaction between the Mo center at the N_2_ molecule. The interaction between charge *q* ^A^ and quadrupole Θ^B^ separated by a distance *R* is given by^27^

1 $$V^{q^{A}\Theta^{B}}=\frac{1}{3}T_{\alpha \beta }q^{A}\Theta_{\alpha \beta }^{B}$$

wherein we used the Einstein summation notation, α and β represent Cartesian coordinates, and

2 $$T_{\alpha \beta }=\frac{\partial }{\partial \alpha }\frac{\partial }{\partial \beta }\frac{1}{R}$$

**Figure 2:**
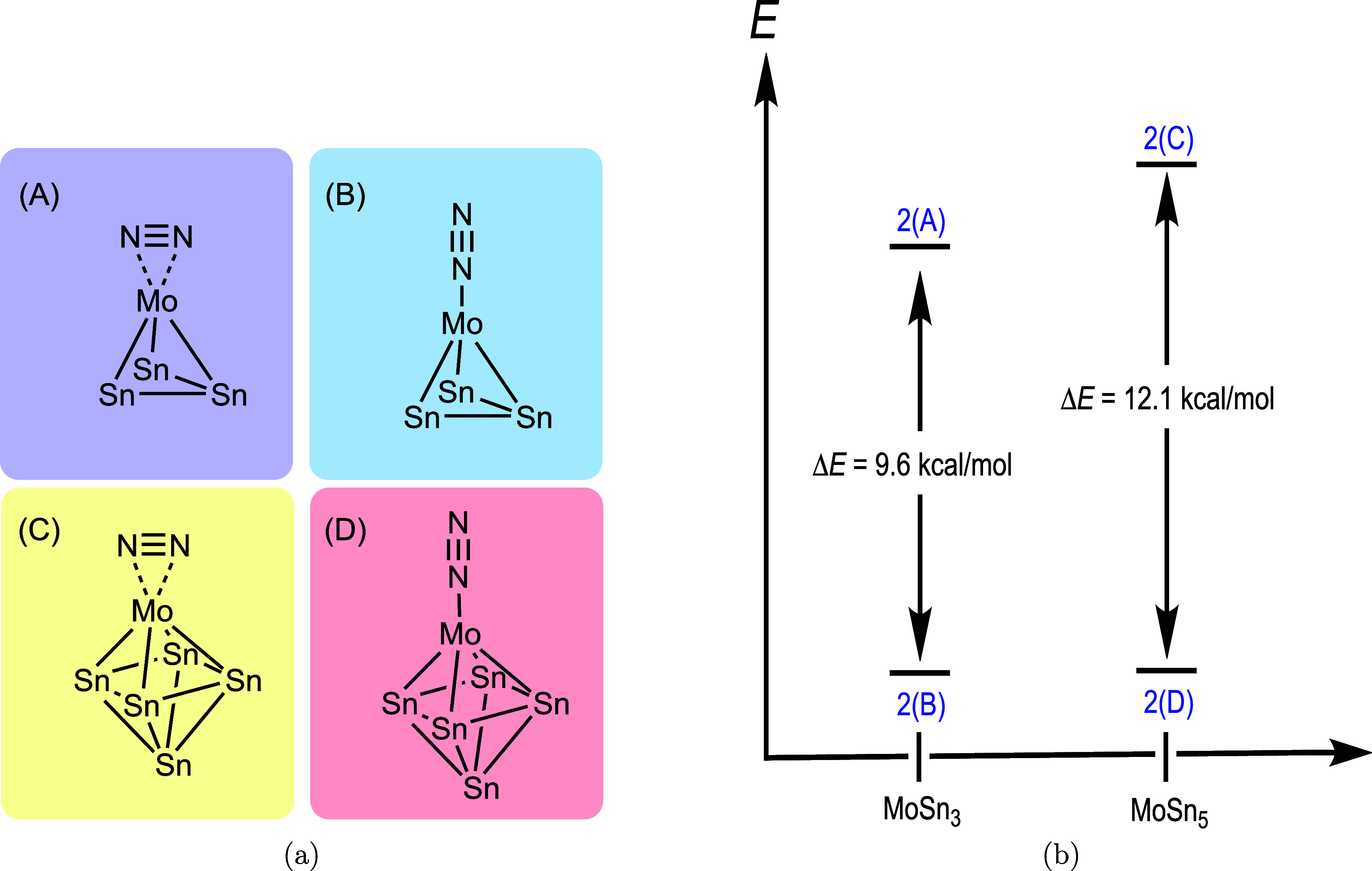
(a) Different conformations for the adsorption of N_2_ on MoSn_3_ (A) and (B) and on MoSn_5_ (C) and (D). (b) Energetic difference for the MoSn_3_ and MoSn_5_ clusters is shown in panel (a).

The consideration of configurations (B) and (D) in Figure 2(a) results in $V^{q^{Mo}\Theta^{N_{2}}}$ = *q* ^Mo^Θ_*zz*_^N_2_^/*R* ^3^, wherein *R* is the distance between the nucleus of the Mo atom and the midpoint of the N_2_ molecule. Because *q* ^Mo^ < 0 in MoSn_3_ and MoSn_5_ ^25^ and $\Theta_{zz}^{N_{2}}<0$ ^28^ configurations (B) and (D) of Figure 2(a) have a repulsive charge–quadrupole interaction between the Mo center and the N_2_ molecule. The same considerations yield an attractive charge–quadrupole interaction for the same species in structures (A) and (C) of Figure 2(a) equal to $V^{q^{Mo}\Theta^{N_{2}}}=-\frac{5}{6}q^{Mo}\Theta_{zz}^{N_{2}}/R^{3}$ . In all cases, adsorption occurs at the Mo center, with Sn atoms acting as structural supports. For MoSn_3_, the structure (B) in Figure 2(a) is 9.6 kcal/mol more stable than its counterpart (A) as schematized in Figure 2(b). Still, both geometries were evaluated as potential precursors for the hydrogenation of N_2_. Ditto for the structures (C) and (D) of the molecular cluster MoSn_5_···N_2_ (Figure 2(a)) with Δ*E* = 12.1 kcal/mol (Figure 2(b)). Despite (i) the above-mentioned charge–quadrupole interactions and (ii) the three-center, two-electron interaction in structures (A) and (C) of Figure 2(a), their respective counterparts (B) and (D) are more stable. We emphasize that the formation of structures (B) (from MoSn_3_ and N_2_) and (D) (from MoSn_5_ and N_2_) involves the generation of the three-center two-electron structures (A) and (C), respectively, as shown in Figure 3. We also note that although structures (B) and (D) are more stable than structures (A) and (C), the formation of structure (C) is a barrierless process and that corresponding to the configuration (A) involves a very low activation energy (>1.0 kcal/mol).

**Figure 3:**
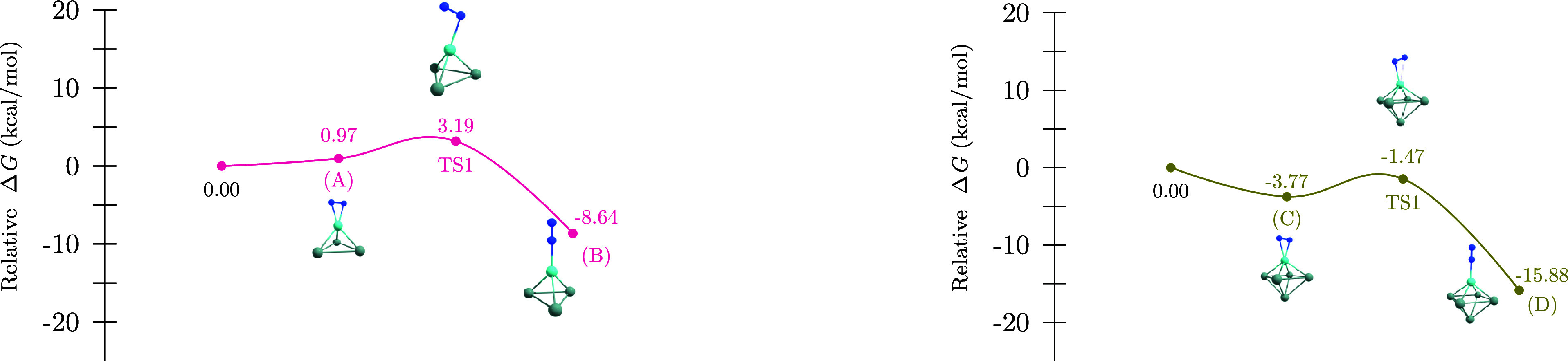
Formation of configurations (B) (left) and (D) (right) from the three-center two-electron structures (A) and (C), respectively. The structures (A–D) are reported in Figure 2.

The three-center, two-electron configurations of the N_2_···MoSn_3_ and N_2_···MoSn_5_ molecular clusters, i.e., structures (A) and (C) in Figure 2, enable hydrogenation pathways, which involve NH–NH and N–NH_2_ moieties. Most importantly, the reaction mechanisms examined herein do not represent the direct N_2_ dissociation of the generally accepted Langmuir–Hinshelwood mechanism^29^ as schematized in the left panel of Figure 1, and they represent alternative reaction pathways for ammonia synthesis. For example, the Mo–N bond in the structures (B) and (D) in Figure 2(a), which is stabilized by the Sn framework, suggests that these clusters directly fix N_2_ via a Mo–N bond and subsequent hydrogenation proceeds at the terminal N atom.

We also investigated the potential for the dissociation of H_2_ on MoSn_3_ and MoSn_5_ clusters. We found low activation barriers for this process, namely, 4.24 and 2.67 kcal/mol for MoSn_3_ and MoSn_5_ clusters, respectively. Therefore, we emphasize that we consider the hydrogen molecules as dissociated and the hydrogen atoms as readily available. This assumption is consistent with the evidence of quantum tunneling by hydrogen atoms^30^ and previously reported observations. For example, on a Pd SAC, Yan and collaborators^31^ found an energy barrier for H_2_ dissociation of 19.37 kcal/mol for the hydrogenation of 1,3-butadiene. Similarly, studies on Fe, Co, and Ni SACs doped in carbon nitride reported dissociation barriers for H_2_ of 8.30, 10.38, and 9.22 kcal/mol, respectively.^32^ The assumption of the availability of atomic hydrogen for the processes investigated herein is also made explicit in Figure 4, which schematizes the three reaction mechanisms explored in this investigation. In line with previous theoretical studies on cluster-mediated nitrogen reduction,^33^ the hydrogenation sequence was analyzed from intermediates containing adsorbed H atoms on the cluster, while the adsorption and dissociation of H_2_ were considered separately as the elementary steps that supply reactive hydrogen to the catalytic cycle. This adsorption of H atoms in the metal cluster is more chemically feasible than their direct interaction with N_2_.^34^

**Figure 4:**
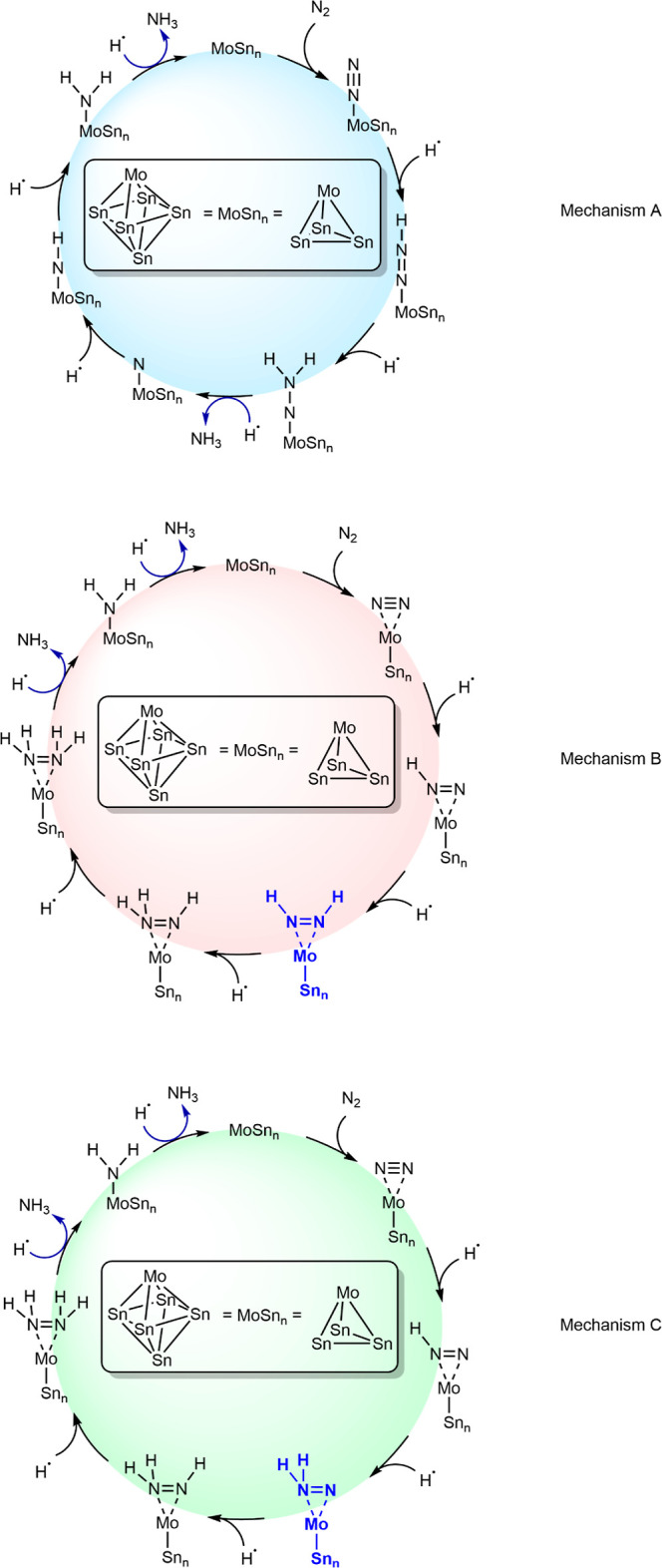
Reaction paths for ammonia synthesis with MoSn_3_ and MoSn_5_ clusters as catalysts. Mechanism A: associative mechanism at the terminal N atom. Mechanisms B and C: associative mechanism at the three-center two-electron adsorption of N_2_. Mechanisms B and C differ in the highlighted structures.

There are different experimental investigations addressing the gas-phase adsorption of N_2_ on metallic clusters, as reported in Table 1. The same chart shows the corresponding adsorption energies as well. We note that the adsorption energies of the clusters investigated herein are overall of smaller magnitude than those of the systems previously examined. We point out, however, that none of the listed, previously reported clusters are electrically neutral. Still, the reported adsorptions for Ta_4_^+^ and CoTaC_2_^–^ are comparable to those computed herein for MoSn_5_···N_2_ in the (D) configuration, as shown in Figure 2. To the best of our knowledge, there are no experimental reports of the adsorption of N_2_ on the MoSn_3_ and MoSn_5_ clusters. Nevertheless, we expect that this study can initiate the development of catalysts with these metals for the activation of N_2_.

**Table 1:** Different Metallic Clusters Addressed in Experimental Investigations of N_2_ Adsorption^a^

metallic cluster	Δ*E*_abs_	references
NbH_2_^–^	–38.97	^35^
NbB_3_O_2_^–^	–18.91	^36^
CoTaC_2_^–^	–10.84	^37^
FeV_2_C_2_^–^	–24.44	^38^
Ta_4_^+^	–17.99	^39^
Mo_5_S_2,3_^+^	–30.67, −32.52	^40^
MoSn_3_	0.97, −8.64	this work
MoSn_5_	–3.77, −15.88	this work

a The corresponding adsorption energies (in kcal/mol) and references are reported as well.

### Reaction Mechanisms

As discussed in the previous section, we identified two stable configurations for each of the MoSn_3_ and MoSn_5_ clusters for nitrogen fixation. We consider three possible mechanisms for nitrogen fixation using these catalysts. Mechanism A (Figure 4) begins when the cluster adsorbs the N_2_ molecule and a nitrogen atom binds to the Mo center, thereby allowing the reaction with hydrogen atoms at the terminal N atom to form the N–NH group. Subsequent hydrogenation steps form the N–NH_2_ moiety and finally the N–NH_3_ structure, weakening the N–N bond with each step until the first NH_3_ molecule is released. These circumstances leave the cluster bound only to the remaining N atom, which is afterward hydrogenated in a similar fashion to the other N atom to form the second ammonia molecule, which subsequently desorbs to recover the cluster and complete the catalytic cycle. Although the enzymatic fixation of N_2_ shown in the left panel of Figure 1 and the reduction of N_2_ to form NH_3_ throughout mechanism A in Figure 4 occur via ionic and free–radical reaction pathways, respectively, we emphasize the similarity between both reaction mechanisms.

Mechanisms B and C follow a sequential hydrogenation governed by the aforementioned three-center, two-electron nitrogen adsorption geometry as shown in Figure 4. The mechanistic difference between these pathways is highlighted in Figure 4, where the sequential hydrogenation can occur either at the same N atom previously hydrogenated, or at the adjacent N atom, forming the adducts with the N–NH_2_ or HN–NH fragments in mechanisms B and C, respectively. The subsequent hydrogenation steps are identical for the remainder of both mechanisms, leading to the formation of H_2_N–NH_2_ groups. Further hydrogenation consecutively forms the two NH_3_ molecules, which leave the cluster to complete the catalytic cycle. We remind the reader that we considered hydrogen atoms readily available and the adsorption and dissociation of H_2_ as elementary steps that supply reactive hydrogen to the catalytic cycle. We considered the catalytic process with MoSn_3_ using molecular H_2_ as the source of hydrogen atoms. We realized that mechanisms B and C are not feasible under these circumstances because the three-center two-electron structure, which underlies these mechanisms is not formed. Figure S6 reports the corresponding Gibbs energy profile for the reduction of N_2_ by considering H_2_ as a source of H atoms and MoSn_3_ as a catalyst. This Gibbs energy profile has a rate-determining step (RDS) with a considerably high activation energy (65 kcal/mol).

#### Mechanism A with MoSn_3_ as Catalyst

We report the Gibbs energy profile of the reaction mechanism A with the MoSn_3_ cluster as catalyst together with the relevant geometries along the reaction coordinate in Figure 5. The corresponding steps are **O** → **A**, it occurs the adsorption of N_2_ in a three-center, two-electron fashion. **A** → **B**, N_2_ rearranges within the cluster to bind only one nitrogen atom to Mo. **B** → **C**, a hydrogen atom is adsorbed on a tin atom to begin the hydrogenation at the terminal nitrogen atom. **C** → **D**, the hydrogen atom of the previous step reacts with the terminal nitrogen to form the moiety N–NH. **D** → **E**, another H atom chemisorbs on a Sn atom of the cluster. **E** → **F**, the hydrogenation of the N–NH moiety yields the N–NH_2_ group. **F** → **G**, an additional H atom is adsorbed on a tin atom. **G** → **H** and **H** → **I**, the formation of the N–NH_3_ fragment takes place. **I** → **J**, the first NH_3_ molecule is released, leaving the cluster bonded to a single nitrogen atom. **J** → **K**, another H atom chemisorbs on a tin center to later bind to the remanent N in the system. **K** → **L**, the formation of the group NH occurs. **L** → **M**, an additional H atom chemisorbs to the Mo center, changing the geometric arrangement of the cluster. **M** → **N**, the hydrogenation of the NH group produces the NH_2_ fragment. **N** → **P**, another H chemisorbs on the Mo center and a Sn atom. **P** → **Q**, the Mo–NH_3_ adduct is formed. **Q** → **R**, the second NH_3_ molecule is released, regenerating the catalyst.

**Figure 5:**
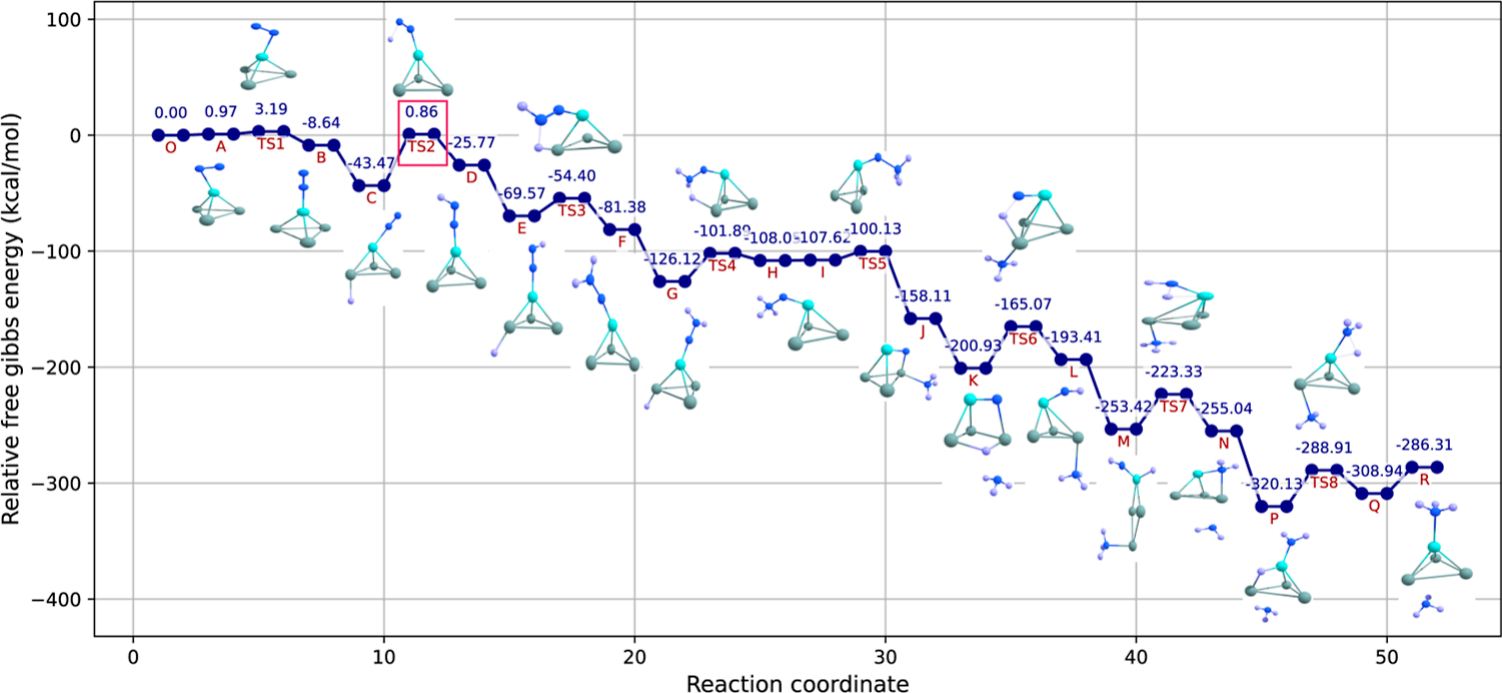
Relative free Gibbs energy profile (in kcal/mol) for mechanism A (Figure 4) for N_2_ fixation with the MoSn_3_ cluster as a catalyst. The steps O–R are described in Section 2.2.1. The number of the transition state associated with the RDS is enclosed in a rectangle, and Figure S7 indicates the evolution of the N–N delocalization index throughout this free Gibbs energy profile in a similar fashion to Figure 13.

The RDS throughout mechanism A using MoSn_3_ as a catalyst corresponds to the first hydrogenation (Δ*G*_act_ = 44.3 kcal/mol), a barrier substantially lower than the industrial HBP (225.86 kcal/mol), which is in agreement with the bond dissociation energy of the N≡N triple bond.

#### Mechanisms B and C in MoSn_3_

Figure 6 shows the Gibbs energy profile for mechanism B with MoSn_3_ as a catalyst along with the corresponding relevant structures. This mechanism consists of the steps: **O** → **A′**, it occurs the adsorption of N_2_ on the cluster via a three-center, two-electron bond and a hydrogen atom binds to the cluster. This atom is adsorbed on both the Mo center and a tin atom. **A′** → **B′**, an adduct with the N–NH moiety is formed. **B′** → **C′**, a hydrogen atom binds to the Mo center on the opposite side to the previous hydrogenation. **C′** → **D′**, the H atom mentioned in the previous step binds to a nitrogen atom, forming the HN–NH fragment. **D′** → **E′**, a hydrogen atom is adsorbed on the cluster, and it forms a chemical bond with Mo. **E′** → **F′**, the H_2_N–NH fragment is formed on the cluster. **F′** → **G′**, another hydrogen atom chemisorbs on a tin atom and the molybdenum center. **G′** → **H′**, an adduct with the H_2_N–NH_2_ fragment is formed. **H′** → **I′**, a hydrogen atom is adsorbed on a tin atom. **I′** → **J′**, the breaking of the N–N bond occurs with no transfer of a previously adsorbed H atom. **J′** → **K′**, both nitrogen atoms become bonded to the Mo atom but on opposite sides of this center. **K′** → **L′**, the adsorbed hydrogen atom binds directly to the closest nitrogen to form the first NH_3_ molecule. **L′** → **M′** and **M′** → **N′**, it occurs the desorption of the first NH_3_ molecule from the cluster. **N′** → **P′**, a hydrogen atom binds to the Mo center and a tin atom. **P′** → **Q′**, an adduct with the second NH_3_ molecule is formed. **Q′** → **R′**, the second NH_3_ molecule is released, regenerating the catalyst.

**Figure 6:**
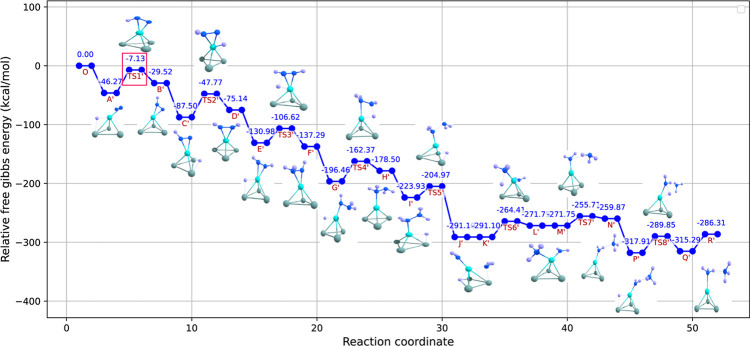
Relative free Gibbs energy profile (in kcal/mol) for mechanism B (Figure 4) for N_2_ fixation with the MoSn_3_ cluster as a catalyst. The steps O–R′ are described in Section 2.2.2. The number of the transition state associated with the RDS is enclosed in a rectangle, and Figure S8 indicates the evolution of the N–N delocalization index throughout this free Gibbs energy profile in a similar fashion to Figure 13.

Likewise, we report the free Gibbs energy profile for mechanism C with MoSn_3_ as a catalyst together with the relevant structures throughout the reaction mechanism in Figure 7. The corresponding steps for this mechanism are **O** → **A′**, it occurs the adsorption of N_2_ on the cluster via a three-center, two-electron bond and a hydrogen atom binds to the cluster. This atom is adsorbed on both the Mo center and a tin atom. **A′** → **B′**, an adduct with the N–NH moiety is formed. **B′** → **C′**, a hydrogen atom binds to the Mo center on the same side to the previous hydrogenation. **C′** → **D′**, the H atom mentioned in the previous step binds to a nitrogen atom, forming the H_2_N–N fragment. **D′** → **E′**, a hydrogen atom is adsorbed on the cluster, and it binds to a Mo center and a tin atom. **E′** → **F′**, the H_2_N–NH fragment is formed on the cluster. **F′** → **G′**, another hydrogen atom chemisorbs on a tin atom and the molybdenum center. **G′** → **H′**, an adduct with the H_2_N–NH_2_ fragment is formed. **H′** → **I′**, an hydrogen atom is adsorbed on a tin atom. **I′** → **J′**, the breaking of the N–N bond occurs with the adsorption of an H atom in the previous step. **J′** → **K′**, both nitrogen atom are bonded to the Mo atoms but on opposite sides of this center. **K′** → **L′**, the adsorbed hydrogen atom binds directly to the closest nitrogen to form the first NH_3_ molecule. **L′** → **M′** and **M′** → **N′**, it occurs the desorption of the first NH_3_ molecule from the cluster. **N′** → **P′**, an hydrogen atom binds to the Mo center and a tin atom. **P′** → **Q′**, an adduct with the second NH_3_ molecule is formed. **Q′** → **R′**, the second NH_3_ molecule is released, regenerating the catalyst.

**Figure 7:**
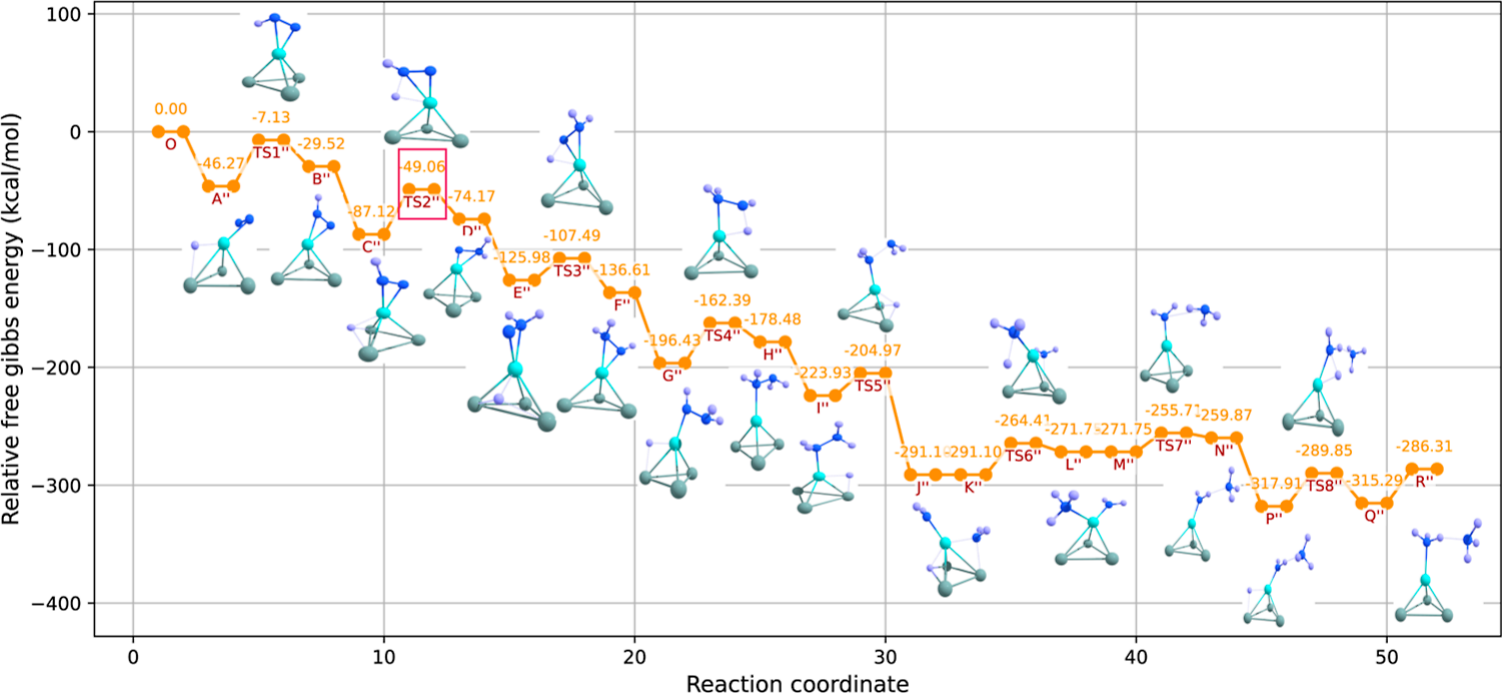
Relative free Gibbs energy profile (in kcal/mol) for mechanism C (Figure 4) for N_2_ fixation with the MoSn_3_ cluster as a catalyst. The steps O–R″ are described in Section 2.2.2. The number of the transition state associated with the RDS is enclosed in a rectangle, and Figure S9 indicates the evolution of the N–N delocalization index throughout this free Gibbs energy profile in a similar fashion to Figure 13.

Mechanisms B and C are energetically similar, as they both involve sequential hydrogenation steps. The subtle difference between these reaction pathways is illustrated in Figure 4, more specifically in the steps **C′** → **D′** (Figure 6) and **C″** → **D″** (Figure 7) with activation barriers of Δ*G*_act_ = 39.7 and Δ*G*_act_ = 38.0 kcal/mol, respectively. The RDS of mechanism B is indeed **C′** → **D′**, while for mechanism C, the RDS is the first hydrogenation step with an activation barrier of Δ*G*_act_ = 39.1 kcal/mol. The major energetic dissimilarity between both mechanisms occurs in the steps **E′** → **F′** and **E″** → **F″** with |ΔΔ*G*_act_| = (5.9 kcal/mol), rendering mechanism C slightly more favorable. In both cases, the reaction results in the stepwise weakening of the N≡N bond via hydrogen additions, as put forward for homogeneous catalysts.^41^ Although mechanism C is slightly favored over mechanism B, the formation of the NH–NH fragment in mechanism B requires a smaller energetic investment (Δ*G*_total_ = 12.4 kcal/mol) than the generation of the N–NH_2_ moiety in mechanism C (Δ*G*_total_ = 13.0 kcal/mol). This result is consistent with the observation that the formation of the NH group is the RDS step in the fixation of N_2_ over Ru nanoparticles in heterogeneous catalysis.^42^ For both mechanisms B and C with the MoSn_3_ as catalyst, the desorption of the second NH_3_ molecule (steps **L′** → **M′** and **L″** → **M″** in Figures 6 and 7, respectively) requires Δ*G*_total_ = 29.0 kcal/mol, i.e., 6.4 kcal/mol than the same process for mechanism A (step **Q** → **R** in Figure 5) for which Δ*G*_total_ = 22.6 kcal/mol.

#### Mechanism A with MoSn_5_

Figure 8 shows the Gibbs energy profile for mechanism A with the MoSn_5_ cluster as a catalyst, along with the relevant structures across the whole process. This reaction mechanism proceeds as follows: **O** → **A**, the adsorption of N_2_ takes place. **A** → **B**, a conformational change occurs, forming a Mo–N bond. **B** → **C**, one hydrogen atom is adsorbed on a tin atom. **C** → **D**, it occurs the formation of the N–NH moiety: the hydrogenation takes place at the terminal nitrogen directly. **D** → **E**, a second hydrogen atom is adsorbed on a tin center of the cluster. **E** → **F**, the N–NH_2_ moiety is formed. **F** → **G**, another hydrogen atom binds to the Mo center and a tin atom of the cluster. **G** → **H** and **H** → **I**, the cleavage of the N–N bond occurs, leaving an N atom alone and the fragment NH_2_ bonded to the Mo center. A conformational change in the cluster also takes place. **I** → **J**, the adsorbed hydrogen atom in the step **F** → **G** migrates to the NH_2_ fragment. The first NH_3_ molecule is formed and it leaves the cluster. **J** → **K**, a hydrogen atom chemisorbs on the Mo center with the N also bonded to the Mo atom. **K** → **L**, the formation of an NH moiety occurs. **L** → **M**, another hydrogen atom chemisorbs onto the Mo center. **M** → **N**, it occurs the formation of the NH_2_ group. **N** → **P**, another hydrogen atom is adsorbed on the Mo center. **P** → **Q**, the formation of the second NH_3_ molecule on the cluster takes place. **Q** → **R**, the catalyst is regenerated with the desorption of the second NH_3_ molecule with Δ*G*_total_ = 34.4 kcal/mol. Mechanism A with MoSn_5_ as catalyst presents the highest RDS of all the mechanisms explored herein from **I** → **J** (Figure 8) with an activation energy Δ*G*_act_ = 69.9 kcal/mol. Mechanisms B and C with MoSn_5_, due to the geometric distortions at the cluster among the tin atoms after the breaking of the N–N bond and the formation of the first NH_3_ molecule.

**Figure 8:**
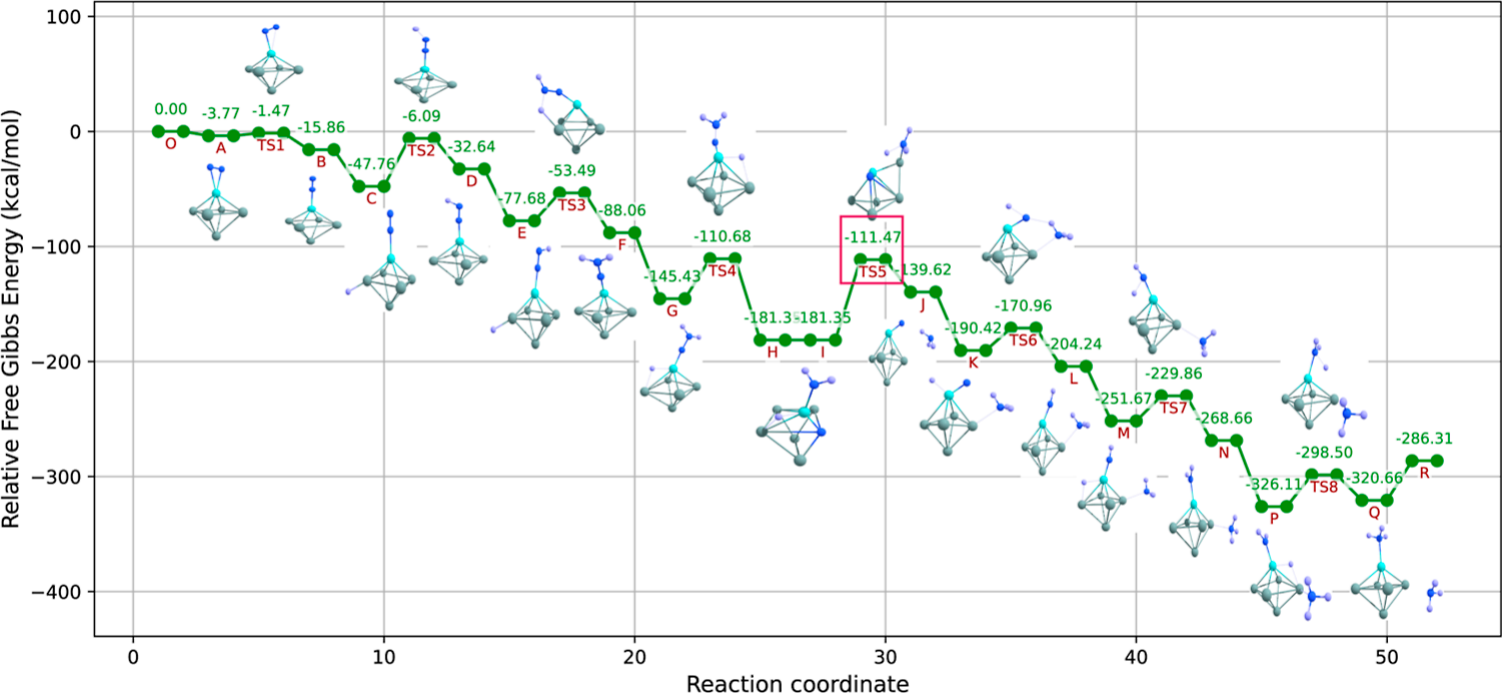
Relative free Gibbs energy profile (in kcal/mol) for mechanism A (Figure 4) for N_2_ fixation with the MoSn_5_ cluster as catalyst. The steps A–R are described in Section 2.2.3. The number of the transition state associated with the RDS is enclosed in a rectangle, and Figure S10 indicates the evolution of the N–N delocalization index throughout this free Gibbs energy profile in a similar fashion to Figure 13.

#### Mechanisms B and C with MoSn_5_

Figure 9 reports the Gibbs energy profile for the fixation of N_2_ via mechanism B in Figure 4 with MoSn_5_ as a catalyst, along with the relevant structures throughout the process. The reaction pathway comprises the steps: **O** → **A′**, it occurs the adsorption of the N_2_ molecule by means of a three-center, two-electron bond and a hydrogen atom is adsorbed on a tin atom and the Mo center. **A′** → **B′**, the formation of the N–NH fragment takes place. **B′** → **C′**, a hydrogen atom is adsorbed on a tin atom of the cluster. **C′** → **D′**, the HN–NH moiety is formed. **D′** → **E′**, another hydrogen atom is adsorbed on a tin atom and the Mo center. **E′** → **F′**, the HN–NH_2_ group is formed. **F′** → **G′**, another hydrogen atom is adsorbed on the Mo atom and on a different Sn center than that referred to in step **B′** → **C′**. **G′** → **H′**, the H_2_N–NH_2_ group is formed on the cluster. **H′** → **I′**, the next hydrogen atom is adsorbed on the Mo center and on another Sn atom. **I′** → **J′**, the first ammonia molecule is released from the cluster. This step is the RDS of the whole process. **J′** → **K′**, the last H atom is adsorbed on the cluster in the same fashion as the previous steps, i.e., on the Mo center and on a tin atom. **K′** → **L′**, the second NH_3_ molecule is formed, elongating the N–Mo bond for its subsequent desorption. **L′** → **M′**, the second ammonia molecule is released, and the catalyst is regenerated.

**Figure 9:**
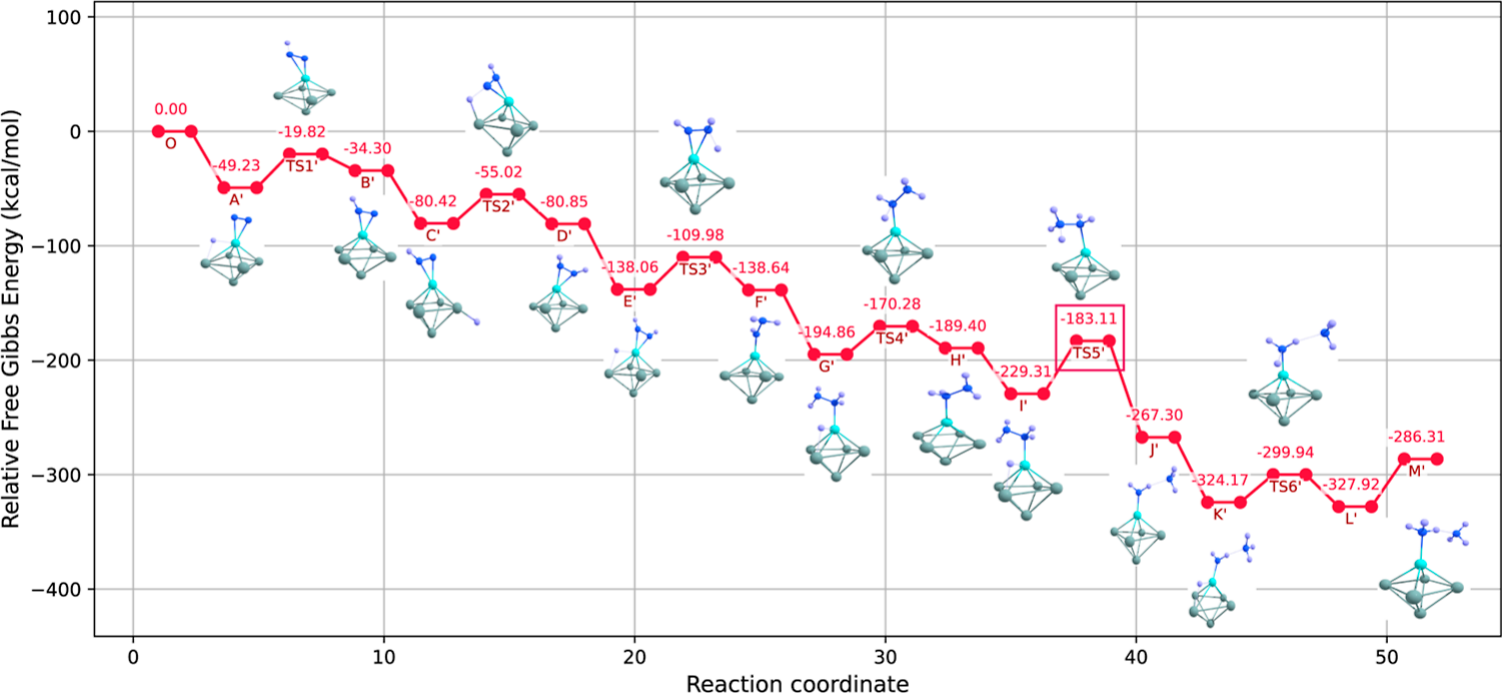
Relative free Gibbs energy profile (in kcal/mol) for mechanism B (Figure 4) for N_2_ fixation with the MoSn_5_ cluster as catalyst. The steps O–M′ are described in Section 2.2.4. The number of the transition state associated with the RDS is enclosed in a rectangle, and Figure S11 indicates the evolution of the N–N delocalization index throughout this free Gibbs energy profile in a similar fashion to Figure 13.

Similarly, the Gibbs energy profile along with the important structures for the fixation of nitrogen via mechanism C (Figure 4) using MoSn_5_ as a catalyst is reported in Figure 10. The corresponding reaction mechanism includes the following steps: **O** → **A″**, the N_2_ molecule is adsorbed in the MoSn_5_ cluster via a three-center, two-electron bond and a hydrogen atom is adsorbed on the Mo center. **A″** → **B″**, it occurs the formation of the N–NH fragment. **B″** → **C″**, an H atom is adsorbed on the Mo center. **C″** → **D″**, the N–NH_2_ group is formed. **D″** → **E″**, a hydrogen atom is adsorbed by a tin atom on the opposite side of the previous adsorption. **E″** → **F″**, the HN–NH_2_ fragment is generated. **F″** → **G″**, another H atom binds to the Mo center. **G″** → **H″**, the H_2_N–NH_2_ moiety is formed. **H″** → **I″**, another hydrogen atom is adsorbed on a Sn atom and on the Mo center. **I″** → **J″**, the first ammonia molecule is released from the cluster. This step is the RDS of the whole process. **J″** → **K″**, the last H atom is adsorbed on the Mo center and on a Sn atom. **K″** → **L″**, the second NH_3_ molecule is formed, elongating the N–Mo bond for its subsequent desorption. **L″** → **M″**, the second ammonia molecule is released, and the catalyst is regenerated.

**Figure 10:**
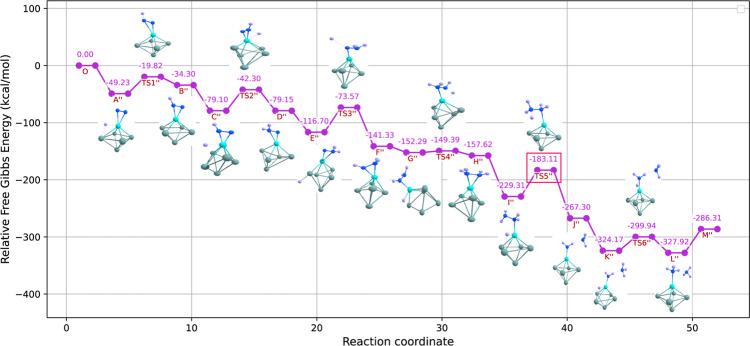
Relative free Gibbs energy profile (in kcal/mol) for mechanism C (Figure 4) for N_2_ fixation with the MoSn_5_ cluster as a catalyst. Relevant structures throughout the complete process are reported as well. The steps O–M″ are described in Section 2.2.4. The number of the transition state associated with the RDS is enclosed in a rectangle, and Figure S12 indicates the evolution of the N–N delocalization index throughout this free Gibbs energy profile in a similar fashion to Figure 13.

Mechanisms B and C, having MoSn_5_ as a catalyst, can be understood as a two-phase mechanism. The first phase involves the sequential hydrogenation of the nitrogen atoms, which leads to the generation of the first ammonia molecule, while the second phase corresponds to the formation of the second ammonia molecule in the remaining N atom. The latter phase is identical for both routes. In the first phase, mechanism B proceeds with hydrogen adsorption on a Sn atom after the initial hydrogenation step, whereas this atom adsorbs on the Mo in mechanism C. At this point, mechanism B continues with a migration of an H atom to form the HN–NH group, leading to **TS2′** in Figure 9. In contrast, mechanism C favors the formation of the H_2_N–N moiety, leading to **TS2″**. The following hydrogenation step of mechanism B requires a higher activation energy compared to mechanism C (43.1 kcal/mol in comparison to 28.1 kcal/mol, respectively). In contrast, the RDS in both mechanisms B and C occurs during the formation of the first NH_3_ molecule (46.2 kcal/mol).

One might think that the choice between MoSn_3_ and MoSn_5_ influences strongly the investigated reaction pathways. Nevertheless, the mechanisms A, B, and C are rather similar for the two examined catalysts. This circumstance is revealed by the overlap of the corresponding Gibbs energy profile which are shown in Figures S3–S5. Concerning the differences for mechanism A with MoSn_3_ and MoSn_5_ as catalysts, we note that the main difference for these reaction pathways correspond to the steps **H** → **I** for which there is an energetic valley for the mechanism with the MoSn_5_ cluster (Figure S3). Throughout this step of the reaction pathway involving the last-mentioned catalyst, the chemical bond between the nitrogen atoms is broken and these atoms remain adsorbed in different parts of the bimetallic cluster, whereas in the mechanism with MoSn_3_, the N–N bond remains even when the first NH_3_ molecule is formed. Mechanism B is very similar for both MoSn_3_ and MoSn_5_ clusters except for the last parts of the reaction pathways starting from step **J′** → **K′**. The reaction pathway with MoSn_5_ has a deeper energetic valley and the mechanism with MoSn_3_ has three extra steps. Another important difference between these reaction mechanisms is the fact that the N–N bond using the MoSn_3_ in mechanism B is broken before a complete formation of the two NH_3_ molecules. The two atoms of nitrogen are adsorbed on different parts of the cluster. This situation does not occur for mechanism B using MoSn_5_ wherein the cleavage of the N–N bond takes places after a complete hydrogenation of the system. The comparison of mechanism C for both catalysts MoSn_3_ and MoSn_5_ is similar to that of mechanism B for these catalysts, i.e., (i) the resemblance of both reaction pathways except for the last part of the mechanism, (ii) the extra steps for the mechanism with MoSn_3_, and (iii) the cleavage of the N–N bond prior to full hydrogenation for the mechanism with MoSn_3_. Along with these differences, the mechanism with MoSn_5_ has a very high activation energy for the formation of **TS3″**. The transition states for these reactions with MoSn_3_ and MoSn_5_ as catalysts are structurally very similar but they are associated to substantially different activation energies.

Among all explored pathways, the mechanisms with MoSn_3_ as a catalyst (in particular mechanism C) exhibit the lowest activation barriers overall, representing the most relevant mechanistic routes for ammonia synthesis investigated herein. Throughout the investigated catalytic cycles, mechanism A with MoSn_5_ as the catalyst displays a very pronounced energy peak compared to the other mechanisms examined in this work, related to a very large deformation of this cluster.

This situation is generally undesirable in any catalytic process. However, all of these barriers remain significantly lower than that of the industrial HBP (225.86 kcal/mol).^2^ The addressed mechanistic routes represent substantial energy savings by fracturing the very large energy barrier of the HBP into smaller, manageable energetic steps.

We additionally consider the migration of hydrogen atoms among the Sn centers in the (A–D) configurations shown in Figure 2. The corresponding reactants and products are almost isoenergetic, and the associated migration processes are essentially barrierless (Table 2) with the exception of the transitions 5 → 4 and 4 → 2 in the MoSn_5_ complex with the (C) configuration, which have barriers for these migrations slightly larger than 11.0 kcal/mol. Because the desorption energy is considerably larger than the migration energy,[Fn fn1] we might consider the Sn framework as a proper hydrogen reservoir.

**Table 2:**
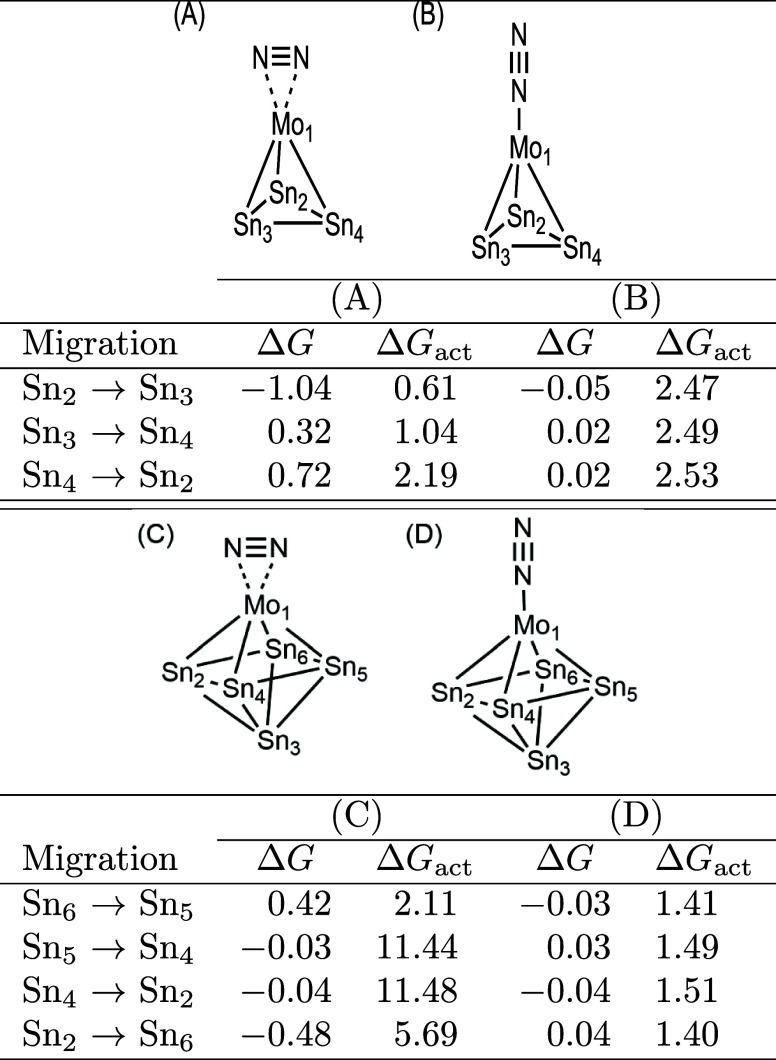
Gibbs Free Energy Changes and Gibbs Free Activation Energies for the Migration of Hydrogen Atoms throughout the Scaffold of Tin Atoms in the Configurations Examined of MoSn_3_ (A) and (B), along with MoSn_5_ (C) and (D)

### Competitive Side Reactions

In principle, the investigated catalysts might experience the following disproportionation reactions

3 $$2{MoSn}_{3}⇌{MoSn}_{2}+{MoSn}_{4}$$

4 $$2{MoSn}_{5}⇌{MoSn}_{6}+{MoSn}_{4}$$

Figure 3 of ref 25 indicates that the clusters MoSn_3_ and MoSn_5_ are energetically stable toward these disproportionation reactions. We considered the disproportionation reactions shown in Figure 11 as possibilities for the poisoning of the addressed catalysts as well. The corresponding Gibbs free energy values are reported in Table 3. Almost all the investigated reactions are thermodynamically spontaneous, with the exception of the disproportionation reaction (b) with MoSn_5_ as catalyst, for which Δ*G*_rxn_ = 11.6 kcal/mol. We point out that these dismutation reactions occurring concomitantly with the investigated reaction mechanisms would yield structures, which either precede or follow the reactants of the disproportionation process across the investigated reaction pathways (Figure 4). The products of this dismutation reaction might be even NH_3_ molecules.

**Figure 11:**
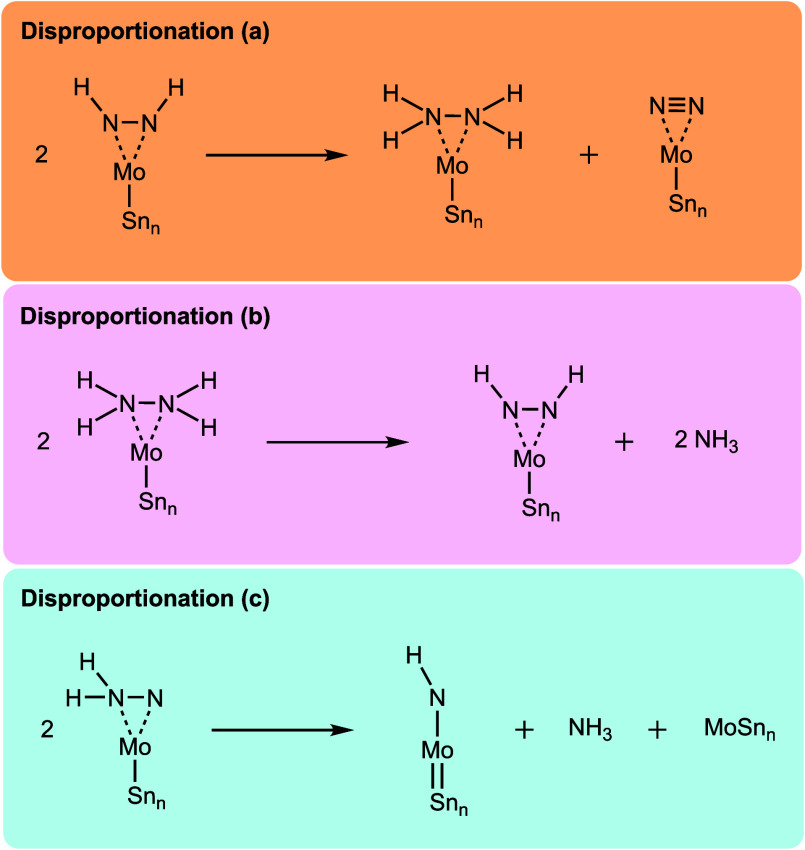
Disproportionation reactions of some intermediaries across the investigated reaction pathways. The labels (a–c) are referred to afterward in Table 3.

**Table 3:** Changes in Gibbs Free Energies for the Different Disproportionation Reactions in Figure 11 with MoSn_3_ and MoSn_5_ as Catalysts^a^

	MoSn_3_	MoSn_5_
disproportionation reaction (a)	–38.8	–43.6
disproportionation reaction (b)	–3.5	11.6
disproportionation reaction (c)	–33.6	–15.6

a The results are reported in kcal/mol.

Likewise, we also explored isomerization reactions with bonded H atoms migrating between the N atoms with both MoSn_3_ and MoSn_5_ clusters as catalysts. The reactions are illustrated in Figure 12, while the activation and reaction energies are reported in Table 4. Similarly to the dismutation reactions above-mentioned, most of the isomerization reactions are thermodynamically spontaneous and for the isomerization reaction (a), having MoSn_3_ as catalyst, we note that Δ*G*_rxn_ = 1.0 kcal/mol. In every case, the isomerization activation energies are smaller than those corresponding to the RDS activation energies for their corresponding mechanisms. We comment further the isomerization reaction (a) with MoSn_5_ as catalyst because it has a very low activation energy (Δ*G*_act_ = 1.3 kcal/mol). In this specific case, the migration of the H atom yields the product of the second hydrogenation of mechanism C and it involves the formation of the N–NH_2_ fragment. The activation energy for the hydrogenation of the product of isomerization (a) having MoSn_5_ as a catalyst has a greater activation than the reactant of the same isomerization reaction with the same catalyst. In the same ways as the disproportionation reactions discussed above, the product of the isomerization just discussed correspond to structures involved in the nitrogen fixation mechanisms proposed in this study. Therefore, both isomerization and disproportionation reactions are not likely to poison the catalysts addressed herein.

**Figure 12:**
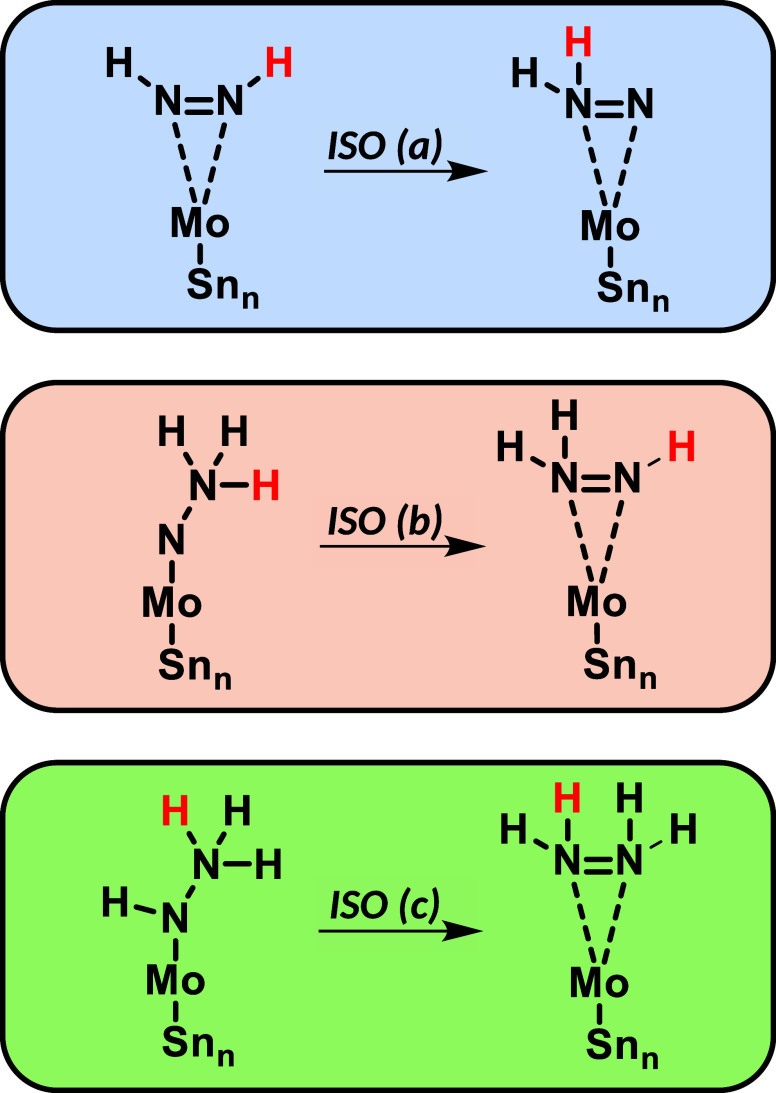
Isomerization reactions investigated for the migration of H atoms between N centers. The labels (a–c) are referred to in the body of the paper and in Table 4.

**Table 4:** Activation and Reaction Energies for the Isomerization Reactions from Figure 12 ^a^

	MoSn_3_		MoSn_5_	
	Δ*G*_act_	Δ*G*_rxn_	Δ*G*_act_	Δ*G*_rxn_
isomerization reaction (a)	47.9	1.0	1.3	–40.0
isomerization reaction (b)	31.1	–29.7	28.3	–25.8
isomerization reaction (c)	32.4	–17.3	34.6	–13.6

a The results are reported in kcal/mol.

We finally consider the potential poisoning of the catalysts due to the formation of Mo–H bonding. There are many steps, which involve the migration of a hydrogen atom from the Mo center to a nitrogen atom, as can be seen in Table 5. Hence, the generation of the Mo–H chemical bond is not likely to poison the catalysts addressed in this investigation.

**Table 5:** Steps in Mechanisms A–C Exploiting both MoSn_3_ and MoSn_5_ as Catalysts for Which There Is a Direct Transfer of an H Atom from the Mo Centre to an N Atom

catalyst	mechanism	elementary steps
MoSn_3_	A	**M** → **N**, **P** → **Q**
	B	**A′** → **B′**, **E′** → **F′**, **G′** → **H′**, **I′** → **J′**, **P′** → **Q′**
	C	**A″** → **B″**, **G″** → **H″**, **I″** → **J″**, **P″** → **Q″**
MoSn_5_	A	**G** → **H**, **K** → **L**, **M** → **N**, **P** → **Q**
	B	**A′** → **B′**, **E′** → **F′**, **G′** → **H′**, **I′** → **J′**, **K′** → **L′**
	C	**A″** → **B″**, **C″** → **D″**, **G″** → **H″**, **I″** → **J″**, **K″** → **L″**

### Wave Function Analysis

#### Chemical Bonding Scenario of the NN Interaction

Now, we consider the change in the chemical bonding scenario between the nitrogen atoms throughout the reaction mechanisms addressed herein. Figure 13 reports the evolution of the DI values between the N centers to assess the degree of covalency of this interaction all through the examined reaction pathways. As shown in the plots displayed in Figure 13(a), the DI tends to decrease progressively along the reaction. The initial value of DI represents the three shared pairs of electrons in the N≡N bond.

**Figure 13:**
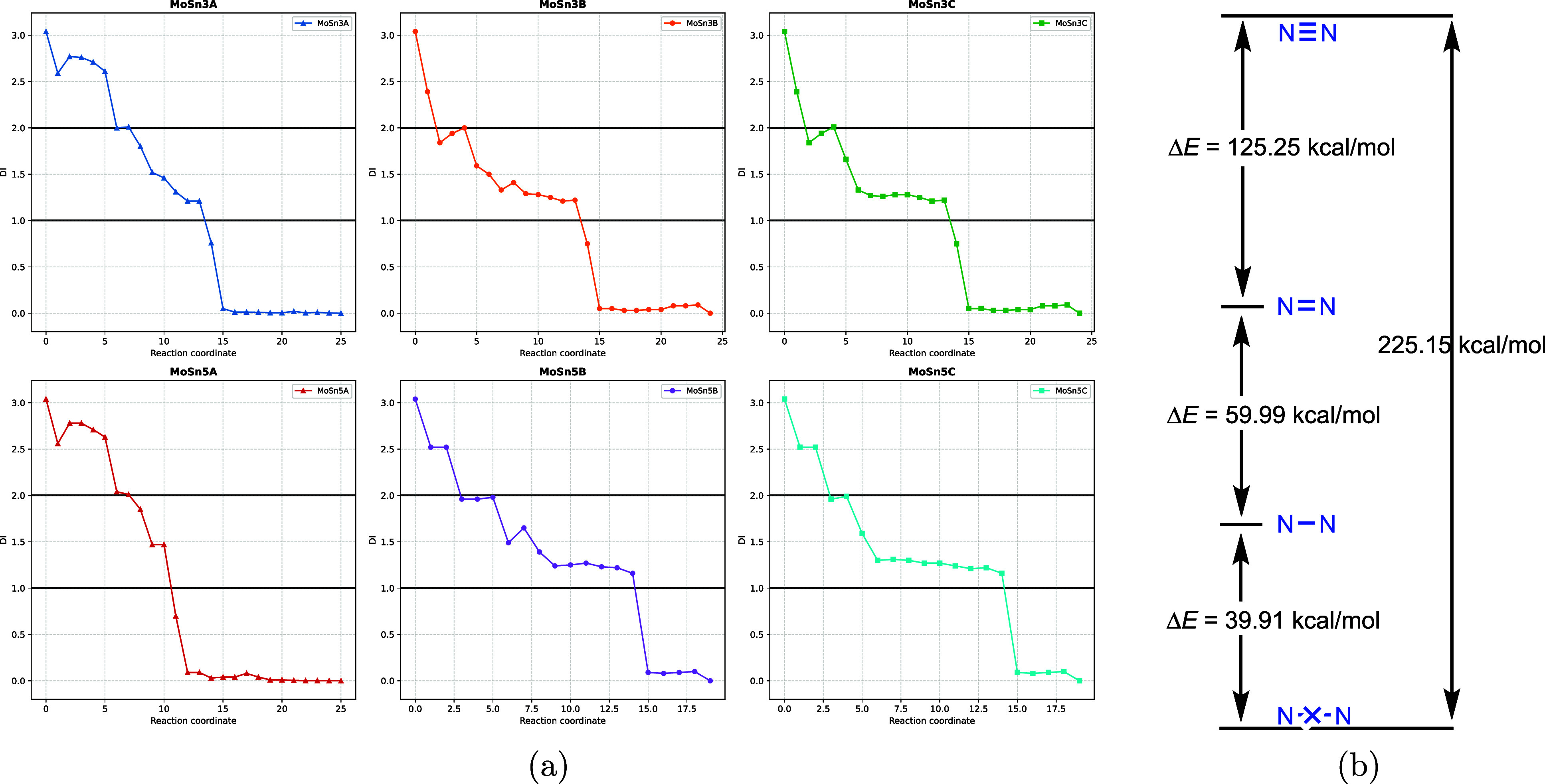
(a) Change in the delocalization index (DI) between the N centers throughout the reaction pathways addressed herein. (b) Successive bond dissociation energies for triple, double, and single nitrogen–nitrogen chemical bonds.

The adsorption of the N_2_ molecule considerably reduces the value of DI from 3 to 2.5, and in the case of mechanisms B and C with MoSn_3_ as a catalyst, the drop is even more pronounced as a hydrogen atom is adsorbed in the metallic cluster as described in steps **A′** → **B′** and **O** → **A″** discussed in Section 2.2.2 and illustrated in Figures 6 and 7. All the reaction pathways exhibit a change in DI from DI ≈ 2.5 to DI ≈ 2 with the occurrence of the first hydrogenation, i.e., the formation of the N–NH moiety. It is at this point of the reaction that the character of the bond between the nitrogen centers changes from triple (N≡N) to double (N=N). We recall that in the breaking of the N≡N bond in a stepwise manner, i.e.,[Fn fn2]

5 N≡N→N=N→N−N→N×N

the most energetically demanding step is the first one, namely, N≡N → N=N, which amounts for roughly 56% of the total binding energy of N≡N (125.25 kcal/mol out of 225.15 kcal/mol) as schematized in Figure 13(b). In other words, the degree of covalency of the N≡N interaction is reduced to approximately 2, which amounts roughly to half of the energy necessary for the breaking of the N≡N bond after the first hydrogenation. It is in this step where the RDS of the reaction occurs for mechanisms A and C with MoSn_3_ as catalyst with activation energies of Δ*G*_act_ = 44.3 and Δ*G*_act_ = 39.1 kcal/mol, respectively. The change in DI from DI ≈ 2 to DI ≈ 1 occurs throughout a series of steps in which those with the most prominent diminution of DI correspond to the second addition of a hydrogen atom to form the N–NH_2_ and NH–NH moieties. Finally, we observed that there is a sudden change from DI ≈ 1 to DI ≈ 0 in all the investigated mechanisms. The reaction steps associated with this transition are those associated with the breaking of the single N–N bond in all the examined reaction pathways. The abrupt character of the change in DI for the nitrogen centers when it goes from DI ≈ 1 to DI ≈ 0 is in agreement with Figure 13b, which indicates that the less energetic step in the sequences of processes (5) is N–N → N×N for which Δ*E* = 39.91 kcal/mol, by virtue of the considerably weak single N–N bond.

#### Chemical Bonding Scenario in the Migration of H Atoms

We consider now the change in the chemical bonding scenario for the migration of hydrogen atoms among the Sn atom framework. In particular, we take into account the changes in the delocalization indices, ΔDI­(Sn, H), throughout the generation of the transition state

6 $$H-{Sn}_{A}-{Sn}_{B}...\overset{\Delta DI{\left({Sn}_{A},H\right)}_{act}}{\underset{\Delta DI{\left({Sn}_{B},H\right)}_{act}}{\to }}{\left[{Sn}_{A}...H...{Sn}_{B}\right]}^{‡}$$

and the overall migration

7 $$H-{Sn}_{A}-{Sn}_{B}...\overset{\Delta DI{\left({Sn}_{A},H\right)}_{total}}{\underset{\Delta DI{\left({Sn}_{B},H\right)}_{total}}{\to }}...{Sn}_{A}-{Sn}_{B}-H$$

Table 6 reports the values of

8 $$\Delta \Delta {DI}_{act}=\Delta DI{\left(Sn_{A},H\right)}_{act}-\Delta DI{\left(Sn_{B},H\right)}_{act}$$

9 $$\Delta \Delta {DI}_{total}=\Delta DI{\left(Sn_{A},H\right)}_{total}-\Delta DI{\left(Sn_{B},H\right)}_{total}$$

as schematized in eqs 6 and 7. In agreement with the small activation and total free Gibbs energies reported in 2, the values of ΔΔDI_act_ are small. This result indicates that the diminution in the delocalization index of the broken Sn_A_–H bond is virtually compensated by the rise of the same quantity of the formed Sn_B_–H bond. In other words, the chemical bond scenario changes slightly throughout the overcoming of the activation barrier for the migration of the H atoms. Likewise, ΔΔD*I*_total_ is small in accordance with the virtually null values of Δ*G* for the overall process.

**Table 6:**
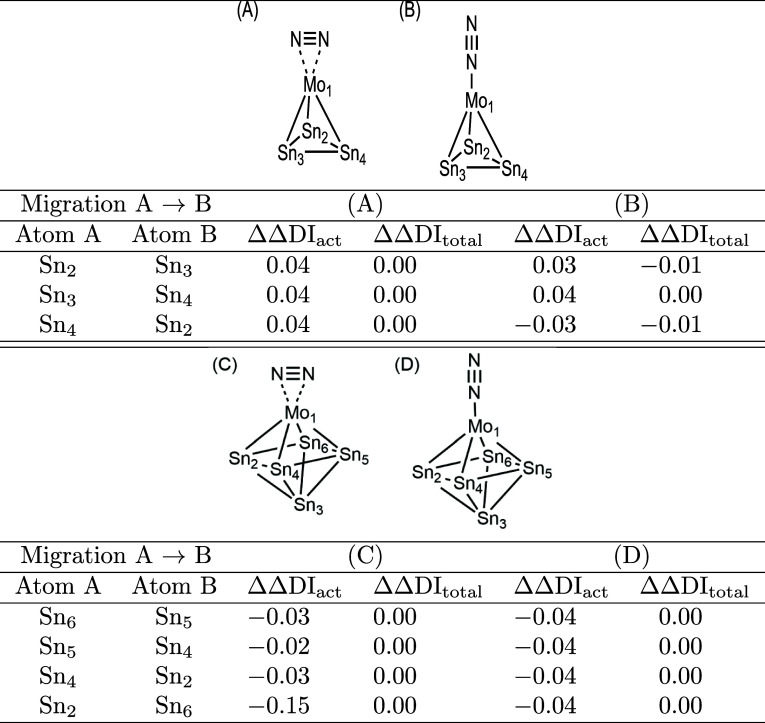
Values of ΔΔDI_act_ and ΔΔD*I*_total_ as Defined in Equations 8 and 9 throughout the Scaffold of Tin Atoms in the Configurations Examined of MoSn_3_ (A) and (B) Together with MoSn_5_ (C) and (D)

#### Chemical Bonding Change in the Rate-Determining Step

We can consider other delocalization indices besides those associated with the interaction between the nitrogen centers. In particular, we examined the values of ΔDI­(RDS), defined as

10 $$\Delta DI\left(RDS\right)=\sum_{A-B}DI\left(A-B\;in\;TS\right)-\sum_{A-B}DI\left(A-B\;in\;reactant\right)$$

i.e., we compared the delocalization values throughout transition states and reactants for RDSs of the reaction mechanisms addressed herein. We considered in eq 10 only those interactions in which the corresponding atoms are linked via a BP[Fn fn3] for either the reactants or the transition state. In general, Table 7 reveals that the larger the absolute value of ΔDI­(RDS), the greater the change in the chemical bonding scenario throughout the RDS and hence the largest the corresponding activation energy.

**Table 7:** Absolute Value of the Change in the QTAIM Delocalization Indexes (ΔDI­(RDS)) as Defined in Equation 10 throughout the Rate Determining Step of the Reaction Mechanisms Investigated Herein^a^

mechanism	|ΔDI(RDS)|	Δ*G*_act_ (kcal/mol)
MoSn_3_ A	0.35	44.33
MoSn_3_ B	0.20	39.74
MoSn_3_ C	0.10	39.13
MoSn_5_ A	1.60	69.88
MoSn_5_ B	0.21	46.19
MoSn_5_ C	0.21	46.19

a The last column reports the activation energy at the RDS in kcal/mol.

On the other hand, Table 8 reports the computed values with the pair of atoms with the most negative changes in DI throughout the RDS for each investigated pathway addressed in this investigation. We considered these pairs of interactions because bond breaking involves (i) a diminution of the covalent character of the interaction, viz., a reduction of the corresponding DI value and (ii) an increase of the energy of the system. A Sn–H interaction has the largest diminution in DI throughout the RDS of mechanism A with MoSn_3_ as a catalyst. Through this RDS, the referred H atom binds to a terminal nitrogen atom of the cluster. This change results in a slight decrease of DI for the N_2_ molecule (ΔDI = −0.10) accompanied by a slight increase in the bond length (Δ*R* = 0.01 Å). In the case of mechanism B with MoSn_3_ as a catalyst, the first hydrogenation at a N atom weakens the N–N bond, increasing their bond distance Δ*R* = 0.02 Å when the hydrogen is adsorbed at the cluster. This adsorption involves the breaking of a three center-two electron configuration and then a further increase of Δ*R* = 0.09 Å. These changes result in a substantial modification of the bond order of the N≡N bond as reported in Table 8 and previously mentioned in the discussion of Figure 13. For mechanism C with MoSn_3_ as a catalyst, the second hydrogenation on the same N atom previously hydrogenated also weakens the N–N bond with ΔDI = −0.35 and Δ*R* = 0.05 Å. This interaction is the most important to account for the observed energetic change in the RDS of this mechanism.

**Table 8:** Two Most Negative Changes in the QTAIM Delocalization Indexes ΔDI at the RDSs for Every Mechanistic Pathway Investigated Herein^a^

pathway	ΔDI	interaction involved
MoSn_3_ A	–0.91	Sn–H
	–0.10	N–N
MoSn_3_ B	–0.55	N–N
	–0.53	Mo–H
MoSn_3_ C	–0.35	N–N
	–0.21	Mo–H
MoSn_5_ A	–0.93	Mo–N
	–0.74	Sn–Sn
MoSn_5_ B	–0.54	Mo–H
	–0.24	Sn–H
MoSn_5_ C	–0.54	Mo–H
	–0.24	Sn–H

a The last column reports the pair of atoms involved.

Regarding the reaction mechanism A having MoSn_5_ as a catalyst, the interaction with the most negative value of ΔDI (ΔDI = −0.93) throughout the RDS is Mo–N, although their distance decreases Δ*R* = −0.21 Å. This circumstance occurs because a hydrogen atom interferes substantially with the Mo–N interaction. Additionally, the geometry of the MoSn_5_ cluster in this RDS changes drastically throughout the formation of the first ammonia molecule. This substantial change in the geometry of the cluster is the main reason in the energetic increase of the system, which occurs to the extent that this mechanism has the highest RDS of all the explored reaction pathways in this investigation. Finally, the DI, which decreases the most for reaction mechanisms B and C with MoSn_5_ as a catalyst, corresponds to the Mo–H interaction. In this case, this step represents the same RDS as shown in Figures 9 and 10. This RDS involves the breaking of the chemical bonding between H and the Mo atom, which is the largest contribution to the increase in energy throughout this elementary step. The energetic cost associated with this chemical bond overwhelms that of the formation of the incipient N–H bond. We emphasize that each pathway exhibits distinct relevant interactions through the RDS, as revealed by the analysis of the contributions to ΔDI­(RDS). These results show that the structure of the MoSn_3_ and MoSn_5_ clusters plays an important role in the examined RDS, and overall, there are drastic geometric changes in the clusters at mechanisms A and C with MoSn_5_, as shown in Figures 8 and 10, resulting in greater activation barriers compared with MoSn_3_, being this cluster more structurally stable with a better performance in the nitrogen fixation process. In particular, the mechanism C having MoSn_3_ as a catalyst has the RDS with the smallest activation energy and the interactions with the less severe changes in the chemical bonding scenario as revealed in the delocalization indices reported in Table 8.

#### QTAIM Charge Analysis of the Mo Center

We conjectured in the Introduction that the fact that the Mo center in both the MoSn_3_ and the MoSn_5_ clusters is negative could aid in the desorption process of the reduced nitrogen atoms. Nevertheless, the molybdenum center loses electronic charge throughout a large part of the examined reaction mechanism, as shown in Figure 14. Indeed, for most of the intermediaries all over the examined reaction mechanisms, the charge of the Mo center is positive, in particular for most of the desorption steps, as shown in Table 9. Therefore, the charge of the Mo center does not play a fundamental role in favoring the desorption of NH_3_ molecules as put forward in the Introduction.

**Figure 14:**
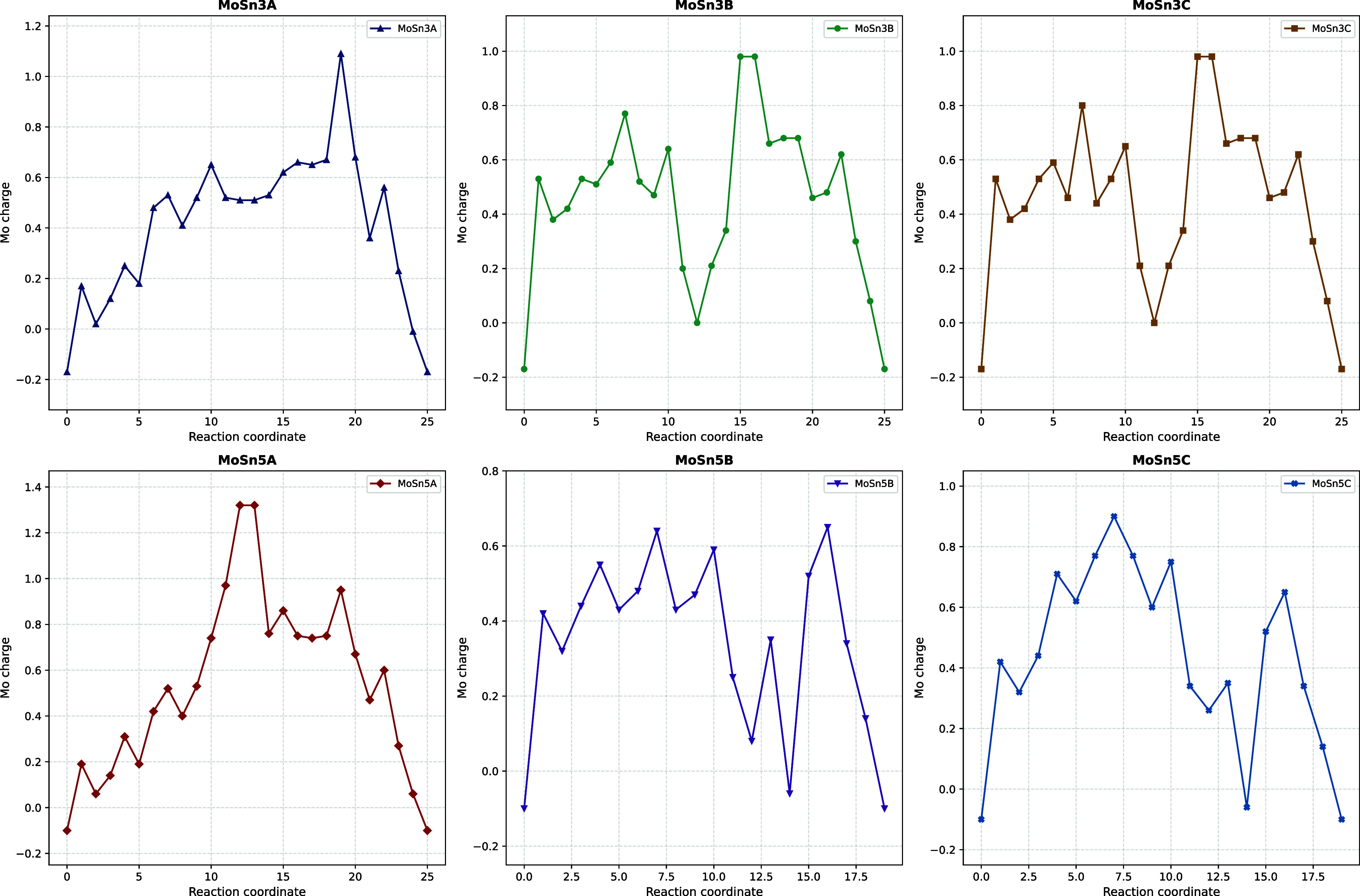
Evolution of the Bader charge of the Mo center throughout the reaction mechanisms A (left), B (center), and C (right) with MoSn_3_ (top) and MoSn_5_ (bottom) as catalysts.

**Table 9:** QTAIM Charges of the Mo Centre and the Nitrogen Atom Bonded to This Metal in the Reactants Corresponding to the First and Second Desorption of NH_3_ Molecules for Every Mechanistic Reaction Pathway Investigated Herein

	first desorption		second desorption	
pathway	*q*(Mo)	*q*(N)	*q*(Mo)	*q*(N)
MoSn_3_ A	0.62	–1.10	–0.01	–1.14
MoSn_3_ B	0.48	–1.28	0.08	–1.20
MoSn_3_ C	0.48	–1.28	0.08	–1.20
MoSn_5_ A	0.86	–0.82	0.06	–1.15
MoSn_5_ B	0.52	–1.26	0.14	–1.21
MoSn_5_ C	0.52	–1.26	0.14	–1.21

#### QTAIM Charge Analysis in the Addition of H Atoms: Formation of Hydrides

We consider now the changes of the QTAIM charge of every adsorbed H atom at the MoSn_3_ and MoSn_5_ clusters. As shown in Table S1 in the Supporting Information, each absorption of an H atom by a metallic center in the cluster resulted in the H atom acquiring a strong hydride character. These hydrides are subsequently transferred to the nitrogen centers directly. This result shows that the MoSn_3_ and MoSn_5_ catalysts mimic the biological behavior of the FeMo-cofactor, which also forms hydrides throughout the nitrogenase catalytic cycle; the FeMo-cofactor accumulates hydrides to carry out all the substrate reductions.^14^ The adsorption of dissociated H atoms onto tin atoms has been previously explored^43^ in a Pt cluster with SnO_2_ as the scaffold, where it was concluded that, once the H_2_ molecule is dissociated, it is advantageous for the reaction to occur that the hydrogen atoms are adsorbed at the surface of the SnO_2_ catalyst. We consider Bader charges for the RDS at the reactant and transition state for the steps with the less activation energy studied herein at MoSn_3_ and MoSn_5_ clusters as representative examples. Figure 15 shows that the reactants of the RDS for mechanisms with MoSn_3_ and MoSn_5_ exhibit a hydride. In the transition state of these steps, the H atom loses electronic density, changing from a hydride to a hydrogen atom in order to bind with a nitrogen center. We can also observe a loss of electronic density for molybdenum and a gain of electron density for the nitrogen atom. Finally, it is important to highlight that, in these steps, the presence of hydrides and the role of tin atoms are crucial, as they actively participate in the hydrogenation of N processes taking place. As previously mentioned, this occurrence of hydrides mimics the activity of the nitrogenase catalytic cycle, as reported by electron–nuclear double resonance (ENDOR) spectroscopy:^14^ “ENDOR spectroscopy has been the key to recognizing the central role of hydrides in the mechanism for nitrogen fixation”, thereby making the catalytic cycle available at milder conditions.

**Figure 15:**
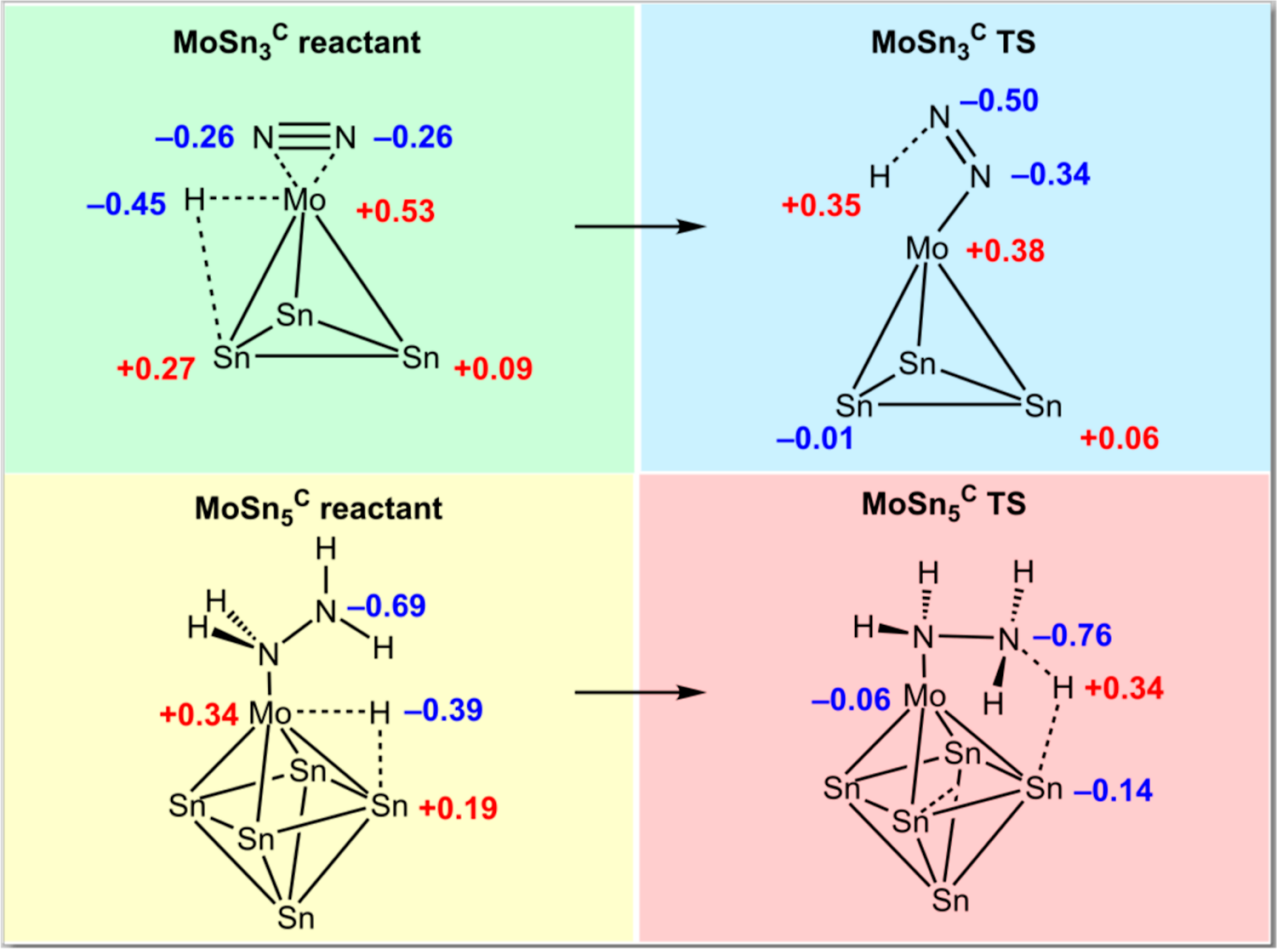
Change in the Bader charge at the reactant and transition state of the RDSs for mechanism C with MoSn_3_ and MoSn_5_ used as catalysts as representative examples.

## Conclusions

We have provided a mechanistic path for the reduction of N_2_ to NH_3_ facilitated by MoSn_3_ and MoSn_5_ clusters by exploring six plausible distinct pathways. We have identified the mechanism C with MoSn_3_ as the most relevant route, having the lowest activation barriers. These catalytic routes reduce the very large energy barrier of the industrial HBP (225.86 kcal/mol) into smaller, manageable energetic steps. Our results highlight the role of molybdenum as an excellent catalytic center and the properties of tin atoms as scaffolds for hydrogen adsorption and subsequent hydrogenation, which together lead to a drastic reduction in the activation barriers. By employing QTAIM analyses, we characterized the chemical bonding scenario and demonstrated that the cluster’s ability to form hydrides mimics the biological activity of the nitrogenase enzyme. The QTAIM analysis elucidated the role of MoSn_3_ and MoSn_5_ in the weakening of the N–N triple bond as well. The QTAIM analyses also showed that the charge of the Mo center is unimportant in the desorption of the resulting NH_3_ molecules. We expect that these results provide valuable insights toward the design of new catalysts on the path to sustainable ammonia production.

## Theoretical Framework

In this section, we discuss briefly the QTAIM method of wave function analysis used in this work. The QTAIM approach allows one to obtain information about the nature of electronic systems via a topological analysis of their electron density ρ­(**r**). Given a normalized approximation of the wave function of an *N*-electron system, Ψ­(**x**_1_, **x**_2_, ..., **x**_*N*_), the expression for its electron density, ρ­(**r**), reads

11 $$\rho \left(r\right)=\left\langle \sum_{i=1}^{N}\delta \left(r-r_{i}\right)\right\rangle =N\int ...\int \Psi \left(x,x_{2},...x_{N}\right){|}^{2}dsdx_{2}dx_{3}...dx_{N}|$$

wherein *s*_*i*_ and **x**_*i*_ denote (i) the spin and (ii) the spatial and spin coordinates of electron *i*. The definition of ρ­(**r**) in eq 11, along with the normalization of the wave function, implies that the electron density is normalized to the number of electrons

12 ∫ρ(r)dr=N

Topological analyses of the electronic density can be performed in terms of critical points (CP) of ρ­(**r**), i.e., points where ∇ρ­(**r**) vanishes. The different types of CP wherein all the curvatures (i.e., the eigenvalues of the Hessian matrix) differ from zero are identified with different elements of molecular structure, namely, nuclei, chemical bonds, rings, and cages.^44^ Each one of these elements of molecular structure is in turn associated to the occurrence of nuclear critical points (NCP), bond critical points (BCP), ring critical points, and cage critical points of the electron density, respectively.

Given the electron density of an *N*-electron system, one can use QTAIM to establish when there is a bond between two atoms A and B. According to QTAIM, there is a bond between atoms A and B when there is a BCP between their nuclei. The unstable manifold of this BCP in the dynamical system defined by ∇ρ­(**r**) comprises two trajectories of ∇ρ­(**r**). One of these trajectories ends in the nucleus of A and the other in the nucleus of B. These trajectories are denoted as the bond path (BP) between atoms A and B. The occurrence of a BCP and a BP linking two nuclei is an indication of the existence of a chemical bond between the corresponding atoms.^45^

The electron density of a molecule or molecular clusters also allows one to divide the 3D space of the system into disjoint regions or atomic basins Ω_A_, Ω_B_, ..., which we identify as the atoms in chemistry.^46^ The space occupied by an atomic basin in QTAIM equals the stable manifold of an NCP. The atoms in QTAIM are divided by interatomic surfaces, i.e., the stable manifold of BCPs. Every point **r** within an interatomic surface satisfies the zero-flux condition

13 ∇ρ(r)·n̂(r)=0

Equation 13 results in the fulfilment of the Schwinger principle for atomic basins.^46^ Therefore, QTAIM atoms are bounded, quantum mechanical systems for which one can compute the expectation values of Dirac observables. For example, the average number of electrons within Ω_A_ is

14 $$\left\langle N\left(\Omega_{A}\right)\right\rangle =\int_{\Omega_{A}}\rho \left(r\right)dr$$

and hence the atomic charge in QTAIM is defined as

15 $$Q^{\Omega_{A}}=Z^{\Omega_{A}}-\left\langle N\left(\Omega_{A}\right)\right\rangle$$

wherein *Z* ^Ω_A_^ is the atomic number of the nucleus inside Ω_A_. One can define in a similar fashion other atomic multipoles, i.e., dipole, quadrupole, etc.^44^

Finally, QTAIM also defines the number of shared electron pairs between two atoms Ω_A_ and Ω_B_, denoted as the delocalization index (DI) between the basins Ω_A_ and Ω_B_ as

16 $$DI\left(\Omega_{A},\Omega_{B}\right)=-2cov\left(N\left(\Omega_{A}\right),N\left(\Omega_{B}\right)\right)$$

in which cov­(*N*(Ω_A_), *N*(Ω_B_)) is the covariance between the random variables *N*(Ω_A_) and *N*(Ω_B_), i.e., the number of electrons in Ω_A_ and in Ω_B_, respectively. The value of DI­(Ω_A_, Ω_B_) establishes the covalent bond order in the QTAIM vision of chemical bonding,^47^ and it aids in the characterization of the covalent component in the interaction between Ω_A_ and Ω_B_.

## Computational Details

All calculations in this study were performed using the PBE0 ^48^ functional along with the D3BJ dispersion correction of Grimme^49,50^ to better assess the noncovalent interactions and the hydrogenation steps of the mechanisms explored herein with the def2-SVP basis set^51^ as implemented in the ORCA 5.0.3 program package.^52–54^ The geometries of the MoSn_3_ and MoSn_5_ clusters, adsorption of N_2_ on the MoSn_3_ and MoSn_5_ clusters, and all nitrogen-fixed intermediates were optimized using this methodology with an energy change convergence tolerance of 1.0 × 10^–6^ *E*_h_ and a maximum gradient convergence tolerance of 1.0 × 10^–4^ *E*_h_/bohr. The PBE0 approximation was chosen for its reliable balance between accuracy and computational efficiency. In particular, the PBE0 functional has been extensively used to describe the interactions between Sn clusters and transition metals.^55^ Relativistic effects were taken into account using the def2-SVP pseudopotentials,^56^ which replace the inner 28 electrons of both Mo and Sn atoms with an effective core potential (ECP)^57,58^ automatically loaded in the ORCA package. The use of ECP to describe relativistic effects has been validated for similar systems in previous studies of metallic clusters.^55^ Transition states were located using two different methodologies. The first employed methodology is (i) the nudged elastic band method (NEB),^59,60^ which, given a reactant and a product that are minima on the PES, finds the first-order saddle point that connects them via a discretized path comprising 12 configurations (images), followed by a transition state search to converge the saddle point. The second exploited methodology is (ii) the quadratic synchronous transit (QST) method,^61,62^ which follows the eigenmode of the interaction of interest (e.g., the bond or angle of the involved hydrogenation step) starting from an initial guess structure. Both methodologies were used with an energy change convergence tolerance of 5.0 × 10^–6^ *E*_h_ and a maximum gradient convergence tolerance of 3.0 × 10^–4^ *E*_h_/bohr. Once the transition state is found, an intrinsic reaction coordinate calculation^63^ was performed to corroborate that the transition state connected the reactants and the products of the corresponding hydrogenation step properly. The choice between NEB and QST depended on the complexity and nature of the reaction step of interest. Numerical frequency analyses were performed to confirm that every transition step found presented only one imaginary frequency. We performed the QTAIM^46^ analysis with the aid of the AIMALL software.^64^ For the generation of suitable electron densities for QTAIM analyses, we performed single-point calculations for each minimum and transition state on every explored pathway using the zero-order regular approximation.^65,66^ These electronic structure calculations were carried out with relativistically contracted orbital and auxiliary basis sets^67^ (SARC/J). Gibbs free energies were computed at 298 K to assess the thermodynamic feasibility of every elementary step in the different investigated reaction pathways.

## Supplementary Material



## Footnotes

a Steps **B** → **C** in Figures 5 and 8 indicate that the corresponding desorption energies of an H atom for MoSn_3_ and MoSn_5_ are 34.83 and 34.90 kcal/mol, respectively.

b The symbol A×B indicates the absence of a chemical bond between atoms A and B.

c See section Theoretical Framework.

## Notes

Extended wave function analyses, relative free Gibbs energy diagrams of MoSn_3_ and MoSn_5_, and corresponding to the intermediaries and transition states addressed in this investigation (PDF)

## References

[1] Erisman J. W., Sutton M. A., Galloway J., Klimont Z., Winiwarter W. (2008). How a century
of ammonia synthesis changed the world. Nat.
Geosci..

[2] Smil V. (1999). Detonator
of the population explosion. Nature.

[3] Haber F. (2002). The synthesis
of ammonia from its elements Nobel Lecture, June 2, 1920. Resonance.

[4] Qian S., Li Z., Sun X., Chen Y., Feng K., Nie K., Yan B., Cheng Y. (2025). Revealing the reaction mechanism of ammonia synthesis
over bimetallic nitride catalyst from a kinetic perspective. Catal. Sci. Technol..

[5] Smith C., Hill A. K., Torrente-Murciano L. (2020). Current and
future role of Haber–Bosch
ammonia in a carbon-free energy landscape. Energy
Environ. Sci..

[6] Nielsen A., Kjaer J., Hansen B. (1964). Rate equation
and mechanism of ammonia
synthesis at industrial conditions. J. Catal..

[7] Ertl G. (1980). Surface science
and catalysis-studies on the mechanism of ammonia synthesis: the P.
H. Emmett award address. Catal. Rev.-Sci. Eng..

[8] Skubic L., Gyergyek S., Huš M., Likozar B. (2024). A review of multiscale
modelling approaches for understanding catalytic ammonia synthesis
and decomposition. J. Catal..

[9] Bozso F. (1977). Interaction
of nitrogen with iron surfaces I. Fe(100) and Fe(111). J. Catal..

[10] Logadóttir A.
´., Nørskov J. K. (2003). Ammonia
synthesis over a Ru(0001)
surface studied by density functional calculations. J. Catal..

[11] Boisen A., Dahl S., Norskov J., Christensen C. (2005). Why the optimal
ammonia synthesis catalyst is not the optimal ammonia decomposition
catalyst. J. Catal..

[12] Yang Z.-Y., Ledbetter R., Shaw S., Pence N., Tokmina-Lukaszewska M., Eilers B., Guo Q., Pokhrel N., Cash V. L., Dean D. R., Antony E., Bothner B., Peters J. W., Seefeldt L. C. (2016). Evidence that the π release
event is the rate-limiting
step in the nitrogenase catalytic cycle. Biochemistry.

[13] Zhao R., Xie H., Chang L., Zhang X., Zhu X., Tong X., Wang T., Luo Y., Wei P., Wang Z., Sun X. (2019). Recent progress in
the electrochemical ammonia synthesis under ambient
conditions. EnergyChem.

[14] Hoffman B. M., Lukoyanov D., Yang Z.-Y., Dean D. R., Seefeldt L. C. (2014). Mechanism
of nitrogen fixation by nitrogenase: the next stage. Chem. Rev..

[15] Humphreys J., Lan R., Tao S. (2021). Development
and recent progress on ammonia synthesis
catalysts for Haber–Bosch process. Adv.
Energy Sust. Res..

[16] Yandulov D. V., Schrock R. R. (2003). Catalytic reduction
of dinitrogen to ammonia at a single
molybdenum center. Science.

[17] Batov M. S., Partlow H. T., Chatelain L., Seed J. A., Scopelliti R., Zivkovic I., Adams R. W., Liddle S. T., Mazzanti M. (2025). Catalytic
and stoichiometric stepwise conversion of side-on bound dinitrogen
to ammonia mediated by a uranium complex. Nat.
Chem..

[18] Li J. (2020). Efficient ammonia electrosynthesis
from nitrate on strained ruthenium
nanoclusters. J. Am. Chem. Soc..

[19] Zeinalipour-Yazdi C. D., Hargreaves J. S. J., Laassiri S., Catlow C. R. A. (2021). A comparative
analysis of the mechanisms of ammonia synthesis on various catalysts
using density functional theory. R. Soc. Open
Sci..

[20] Zeinalipour-Yazdi C. D., Hargreaves J. S. J., Catlow C. R. A. (2018). Low-T mechanisms
of ammonia synthesis
on Co_3_Mo_3_N. J. Phys. Chem.
C.

[21] Qiao B., Wang A., Yang X., Allard L. F., Jiang Z., Cui Y., Liu J., Li J., Zhang T. (2011). Single-atom catalysis
of CO oxidation using Pt_1_/FeO_x_. Nat. Chem..

[22] Yang X.-F., Wang A., Qiao B., Li J., Liu J., Zhang T. (2013). Single-atom catalysts: a new frontier
in heterogeneous catalysis. Acc. Chem. Res..

[23] Ma X.-L., Liu J.-C., Xiao H., Li J. (2018). Surface single-cluster
catalyst for N_2_ to NH_3_ thermal conversion. J. Am. Chem. Soc..

[24] Zhang S., Zong L., Zeng X., Zhou R., Liu Y., Zhang C., Pan J., Cullen P. J., Ostrikov K. K., Shao T. (2022). Sustainable nitrogen
fixation with nanosecond pulsed spark discharges:
insights into free-radical-chain reactions. Green Chem..

[25] Guevara-Vela J. M., Gallegos M., Francisco E., Martín Pendás A. ´., Rocha-Rinza T. (2025). Structural
evolution and bonding within molybdenum-doped
tin clusters, MoSn_n_ (n = 2 – 15). Inorg. Chim. Acta.

[26] Bora D., Gayen F. R., Saha B. (2022). Ammonia from
dinitrogen at ambient
conditions by organometallic catalysts. RSC
Adv..

[27] Stone, A. The Theory of Intermolecular Forces; Oxford University Press, 2013

[28] Halkier A., Coriani S., Jørgensen P. (1998). The molecular
electric quadrupole
moment of N_2_. Chem. Phys. Lett..

[29] Sajid M., Kaden W. E., Kara A. (2022). DFT investigation
of ammonia formation
via a Langmuir–Hinshelwood mechanism on Mo-terminated δ-MoN(0001). ACS Omega.

[30] Hand M. R., Holloway S. (1989). A theoretical study
of the dissociation of H_2_/Cu. J.
Chem. Phys..

[31] Yan H., Lv H., Yi H., Liu W., Xia Y., Huang X., Huang W., Wei S., Wu X., Lu J. (2018). Understanding
the underlying mechanism of improved selectivity in Pd_1_ single-atom catalyzed hydrogenation reaction. J. Catal..

[32] Shah A. B., Sarfaraz S., Yar M., Sheikh N. S., Hammud H. H., Ayub K. (2023). Remarkable single atom
catalyst of transition metal (Fe, Co &
Ni) doped on C_2_N surface for hydrogen dissociation reaction. Nanomaterials.

[33] Cui C., Zhang H., Cheng R., Huang B., Luo Z. (2022). On the nature
of three-atom metal cluster catalysis for N_2_ reduction
to ammonia. ACS Catal..

[34] Ling C., Zhang Y., Li Q., Bai X., Shi L., Wang J. (2019). Jinlan New mechanism for N_2_ reduction: the
essential role
of surface hydrogenation. J. Am. Chem. Soc..

[35] Wang M., Chu L.-Y., Li Z.-Y., Messinis A. M., Ding Y.-Q., Hu L., Ma J.-B. (2021). Dinitrogen and carbon
dioxide activation to form C–N
bonds at room temperature: a new Mechanism revealed by experimental
and theoretical studies. J. Phys. Chem. Lett..

[36] Wang M., Zhou H.-Y., Messinis A. M., Chu L.-Y., Li Y., Ma J.-B. (2021). Nitrogen activation
and transformation on monometallic niobium boron
oxide cluster anions at room temperature: a dual-site mechanism. J. Phys. Chem. Lett..

[37] Mou L.-H., Li Y., Wei G.-P., Li Z.-Y., Liu Q.-Y., Chen H., He S.-G. (2022). Mutual functionalization of dinitrogen and methane mediated by heteronuclear
metal cluster anions CoTaC^–^
_2_. Chem. Sci..

[38] Mou L.-H., Li Y., Li Z. Y., Liu, Yu Q., Ren Yi, Chen H., He S.-G. (2020). Dinitrogen activation and functionalization
by heteronuclear metal
cluster anions Ta^+^
_4_ at room remperature. J. Phys. Chem. Lett..

[39] Gao Y., Cheng R., Hansen K., Luo Z. (2026). Dual activation of
CO_2_ and N_2_ facilitated by single Ta^+^
_4_ clusters. Chem. Sci..

[40] Jiang G.-D., Yang Q., Wei G.-P., Li Z.-Y., He S.-G. (2023). Superior
reactivity of molybdenum–sulfur cluster anions Mo_5_S^2–^ and Mo_5_S^–^
_3_ toward dinitrogen. Inorg. Chem..

[41] Schrock R. (2008). Catalytic
reduction of dinitrogen to ammonia by molybdenum: theory versus experiment. Angew. Chem., Int. Ed..

[42] Hara M., Kitano M., Hosono H. (2017). Ru-loaded C_12_A7:e^–^ electride as a catalyst for ammonia synthesis. ACS Catal..

[43] Zyubina T. S., Zyubin A. S., Dobrovol’skii Y. A., Volokhov V. M., Arsatov A. V., Bazhanova Z. G. (2011). Dissociative
adsorption of molecular
hydrogen onto Pt_6_ and Pt_19_ platinum clusters
located on the tin dioxide surface: quantum-chemical modeling. Russ. J. Inorg. Chem..

[44] Bader, R. F. W. Atoms in Molecules: A Quantum Theory; Oxford University Press, 1990

[45] Bader R. F.
W. (1998). A bond
path: a universal indicator of bonded interactions. J. Phys. Chem. A.

[46] Bader R. F.
W. (2010). Definition
of molecular structure: by choice or by appeal to observation?. J. Phys. Chem. A.

[47] Fradera X., Austen M. A., Bader R. F. W. (1999). The
Lewis model and beyond. J. Phys. Chem. A.

[48] Adamo C., Barone V. (1999). Toward reliable density functional methods without
adjustable parameters: the PBE0 model. J. Chem.
Phys..

[49] Grimme S., Antony J., Ehrlich S., Krieg H. (2010). A consistent and accurate
ab-initio parametrization of density functional dispersion correction
(DFT-D) for the 94 elements H–Pu. J.
Chem. Phys..

[50] Grimme S., Ehrlich S., Goerigk L. (2011). Effect of the damping function in
dispersion corrected density functional theory. J. Comput. Chem..

[51] Weigend F., Ahlrichs R. (2005). Balanced basis sets
of split valence, triple zeta valence
and quadruple zeta valence quality for H to Rn: design and assessment
of accuracy. Phys. Chem. Chem. Phys..

[52] Neese F. (2012). The ORCA program
system. WIREs Comput. Mol. Sci..

[53] Neese F. (2022). Software update:
The ORCA program system-Version 5.0. WIREs Comput.
Mol. Sci..

[54] Neese F., Wennmohs F., Becker U., Riplinger C. (2020). The ORCA quantum
chemistry program package. J. Chem. Phys..

[55] Lehr A., Jäger M., Gleditzsch M., Rivic F., Schäfer R. (2020). Optical absorption
of atomically-precise Sn_14_ nanoclusters: the antagonistic
interplay of ligand stabilization, molecular symmetry, and solvatochromism. J. Phys. Chem. Lett..

[56] Weigend F., Baldes A. (2010). Segmented contracted basis sets for one- and two-component
Dirac–Fock effective core potentials. J. Chem. Phys..

[57] Dolg M., Stoll H., Preuss H. (1989). Energy-adjusted
ab-initio pseudopotentials
for the rare earth elements. J. Chem. Phys..

[58] Metz B., Stoll H., Dolg M. (2000). Small-core multiconfiguration-Dirac–Hartree–Fock-adjusted
pseudopotentials for post-d main group elements: application to PbH
and PbO. J. Chem. Phys..

[59] Jónsson, H. ; Mills, G. ; Jacobsen, K. W. Nudged elastic band method for finding minimum energy paths of transitions. Classical and Quantum Dynamics in Condensed Phase Simulations; World Scientific, 1998; pp 385–404.

[60] Ásgeirsson V., Birgisson B. O., Bjornsson R., Becker U., Neese F., Riplinger C., Jónsson H. (2021). Nudged elastic band method for molecular
reactions using energy-weighted springs combined with eigenvector
following. J. Chem. Theory Comput..

[61] Baker J. (1986). An algorithm
for the location of transition states. J. Comput.
Chem..

[62] Harvey J. N., Aschi M., Schwarz H., Koch W. (1998). The singlet and triplet
states of phenyl cation. A hybrid approach for locating minimum energy
crossing points between non-interacting potential energy surfaces. Theor. Chem. Acc..

[63] Ishida K., Morokuma K., Komornicki A. (1977). The intrinsic
reaction coordinate.
An ab initio calculation for HNC → HCN and H^–^ + CH_4_ → CH_4_ + H^–^. J. Chem. Phys..

[64] Keith, T. A. AIMAll, (Version 19.02.13), TK Gristmill Software: Overland Park KS, USA, 2019, aim.tkgristmill.com.

[65] Lenthe E. v., Baerends E. J., Snijders J. G. (1993). Relativistic regular
two-component
Hamiltonians. J. Chem. Phys..

[66] Pantazis D. A., Chen X.-Y., Landis C. R., Neese F. (2008). All-Electron scalar
relativistic basis sets for third-Row transition metal atoms. J. Chem. Theory Comput..

[67] Rolfes J. D., Neese F., Pantazis D. A. (2020). All-electron
scalar relativistic
basis sets for the elements Rb–Xe. J.
Comput. Chem..

