# Supplementary material for: Nitrogen Fixation by MoSn3 and MoSn5 Clusters
Source: ACS Omega. 2026 Jul 24;11(31):46154–71. doi: 10.1021/acsomega.6c02579 (PMC13470869; doi:10.1021/acsomega.6c02579)
Supplement: Supplementary file 1 [file ao6c02579_si_001.pdf]

Nitrogen fixation by $MoSn_3$ and $MoSn_5$ clusters

Jesús Iván Salazar-Barrientos[1], Thamara V. Salazar-Barrientos[2], Peter T. Cummings[3], José Manuel Guevara-Vela*[3], and Tomás Rocha-Rinza*[1]

[1] *Instituto de Química, Universidad Nacional Autónoma de México, Circuito Exterior, Ciudad Universitaria s/n, Delegación Coyoacán, CP 04510, Ciudad de México, México.*
[2] *Canadian Centre for Research in Advanced Fluorine Technologies (C-CRAFT) and Department of Chemistry and Biochemistry, University of Lethbridge, Lethbridge, T1K 3M4, Alberta, Canada*
[3] *School of Engineering and Physical Sciences, Heriot-Watt University, Edinburgh EH14 4AS, Great Britain.*

*j.guevara-vela@hw.ac.uk, trocha@iquimica.unam.mx

Supporting Information

Relative Energy Diagrams

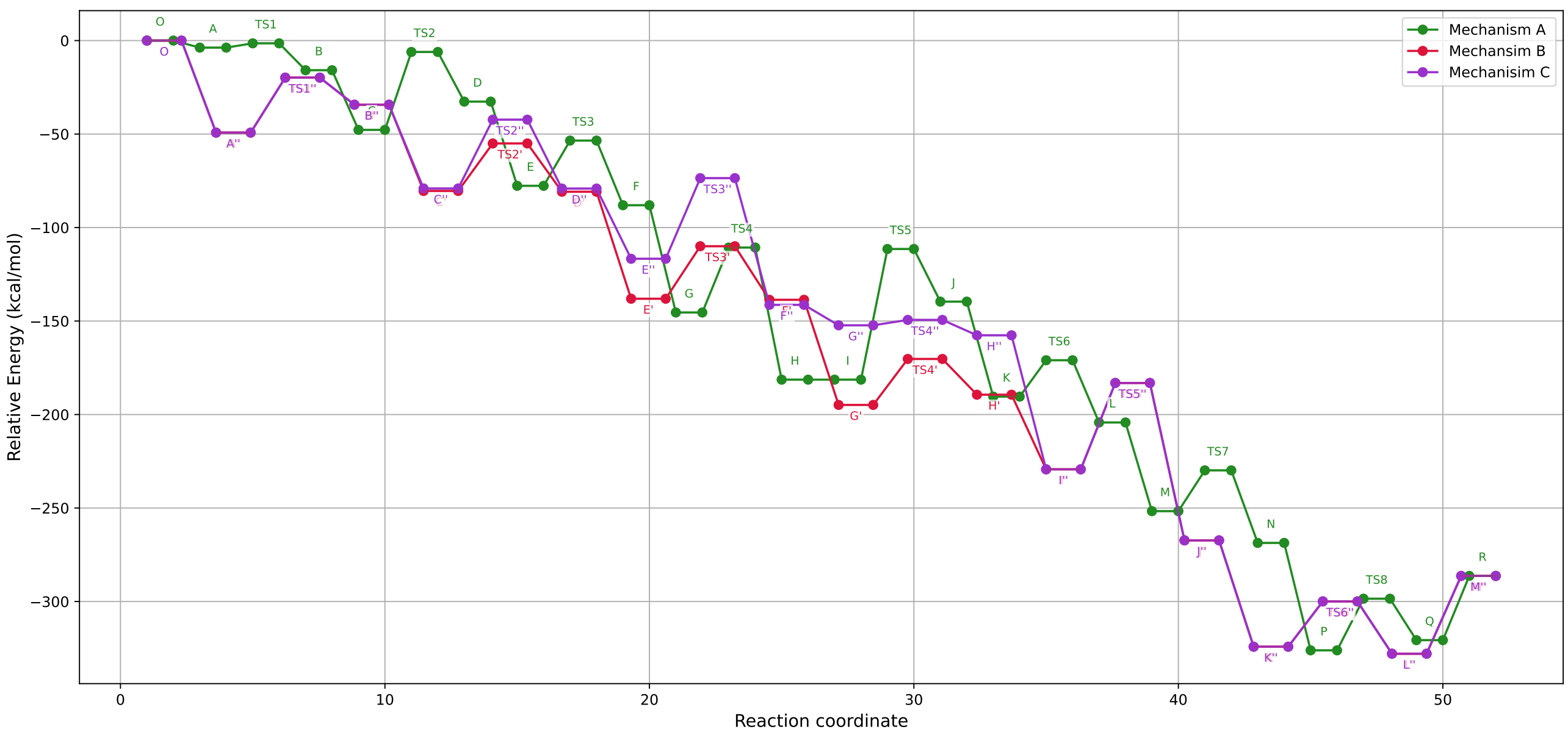

Figure S1: Relative Gibbs free energy diagrams for all the $MoSn_5$ mechanisms explored in this investigation.

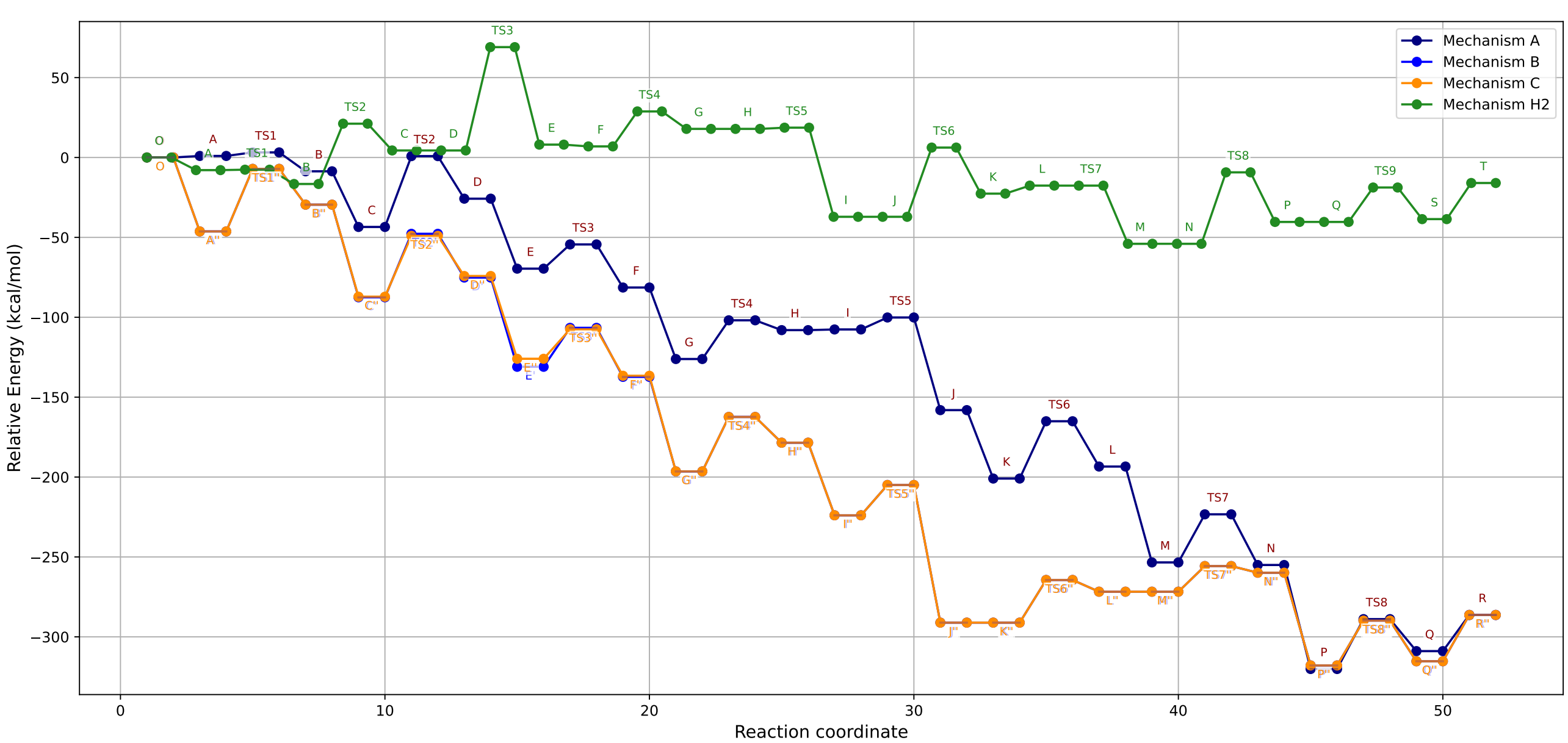

Figure S2: Relative Gibbs free energy diagrams for all the $MoSn_3$ mechanism explored in this investigation.

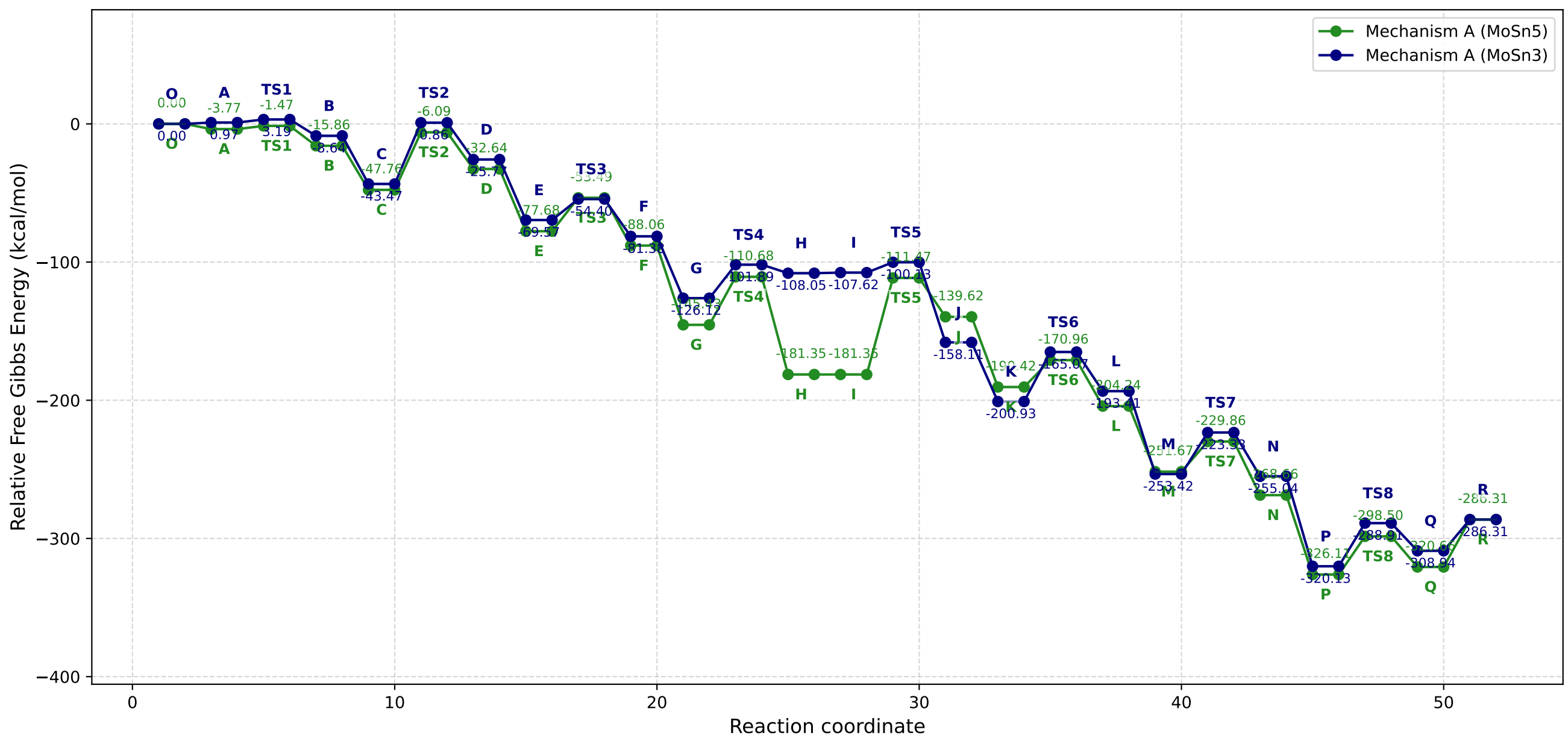

Figure S3: Relative Gibbs free energy diagrams for mechanism A with $MoSn_3$ and $MoSn_5$ as catalysts.

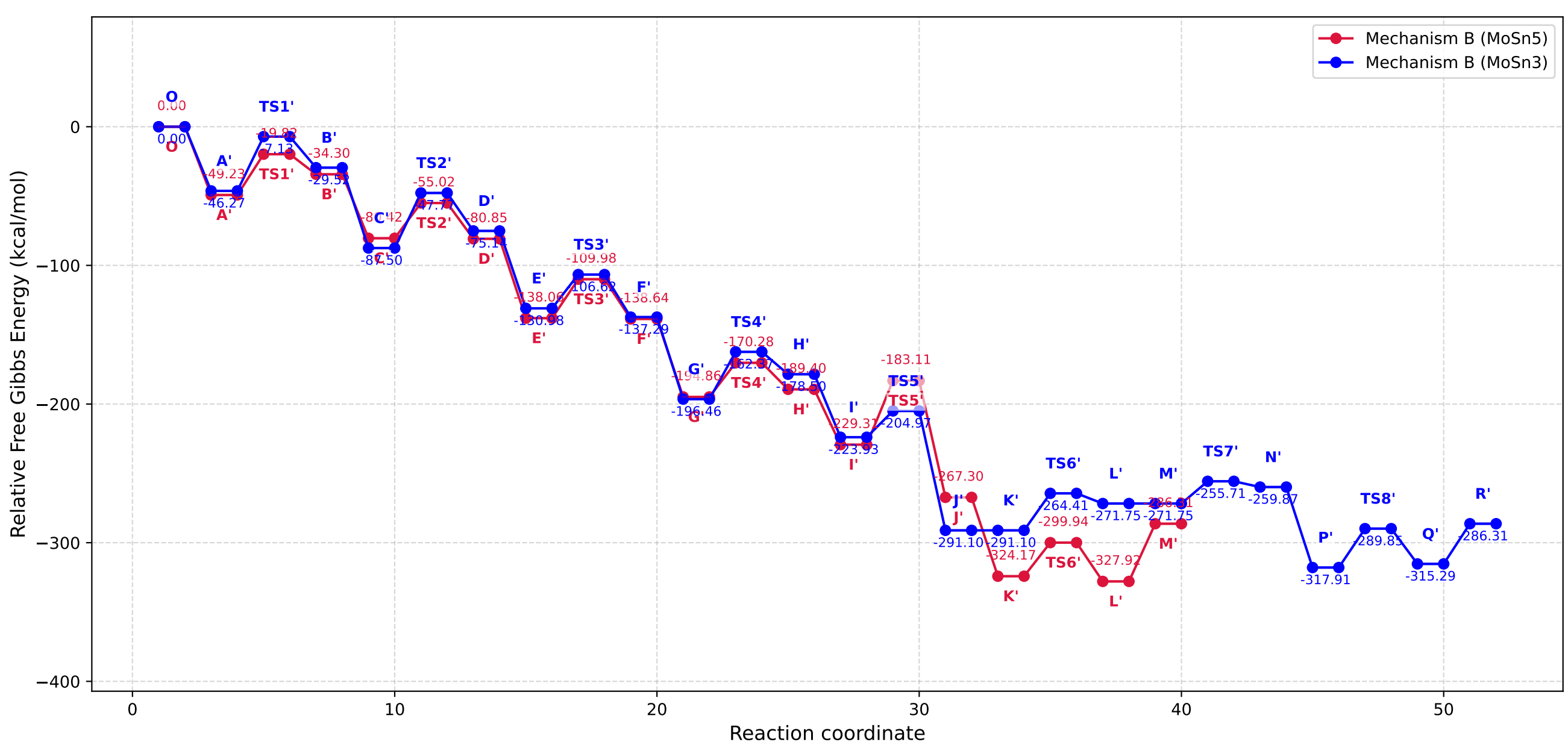

Figure S4: Relative Gibbs free energy diagrams for mechanism B with $MoSn_3$ and $MoSn_5$ as catalysts.

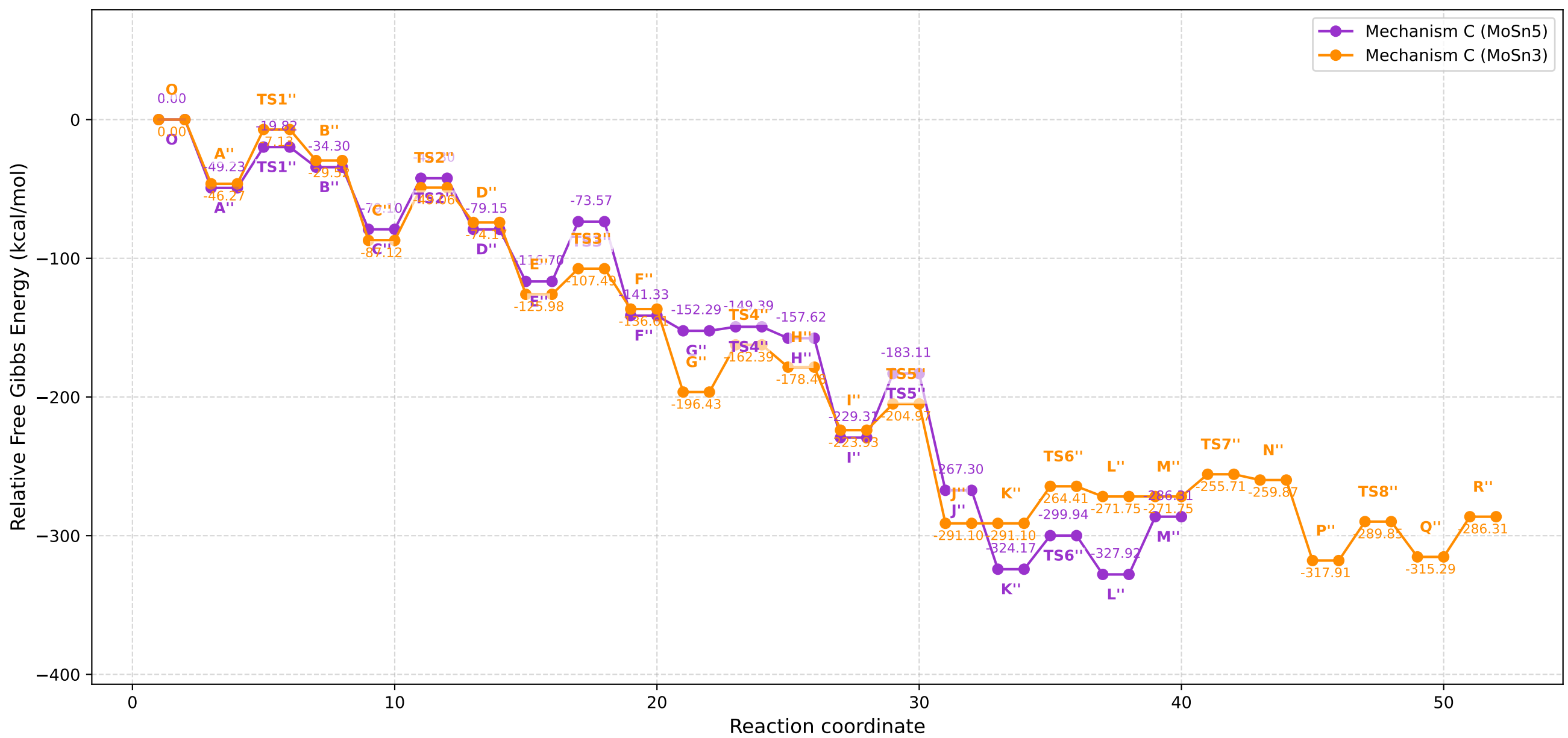

Figure S5: Relative Gibbs free energy diagrams for mechanism C with $MoSn_3$ and $MoSn_5$ as catalysts.

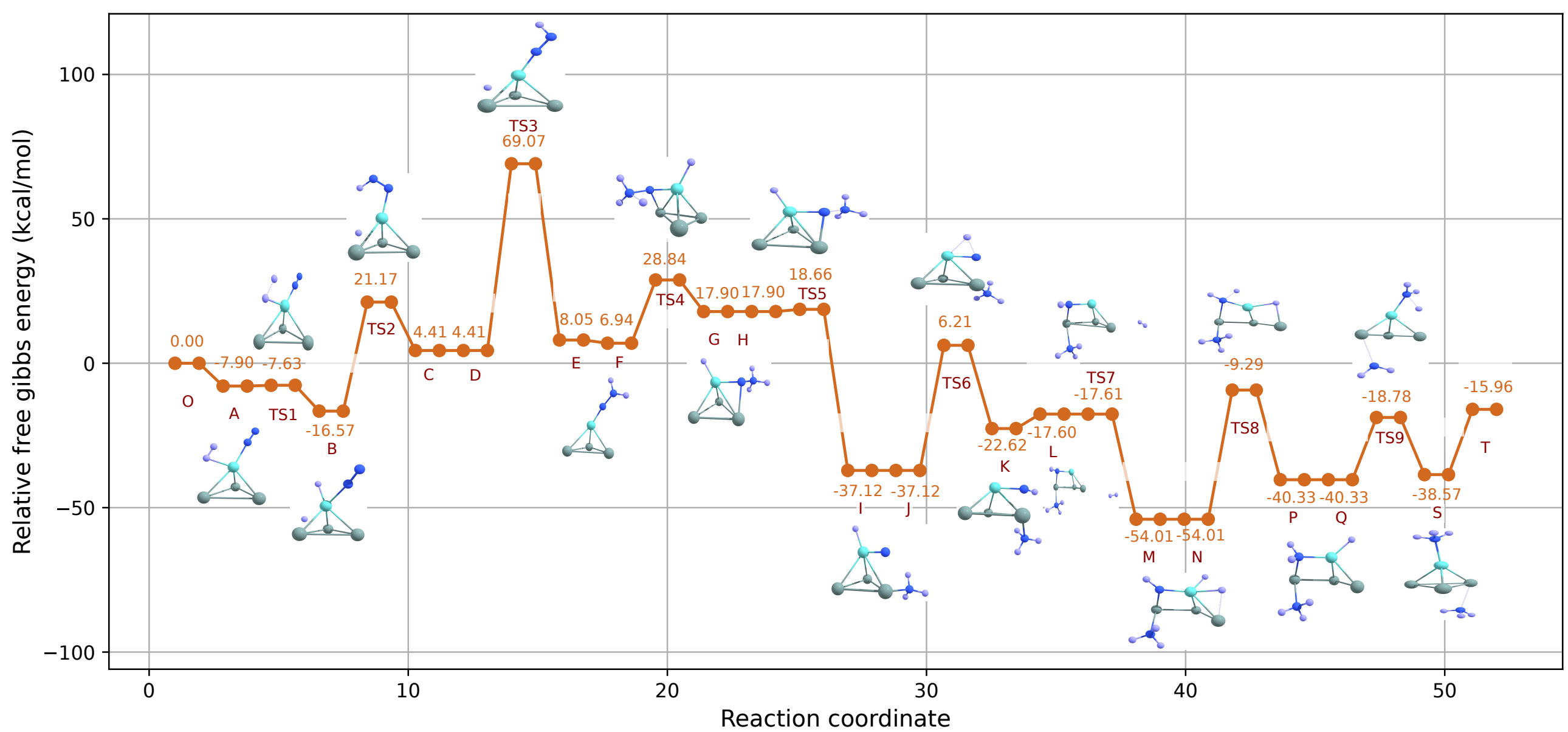

Figure S6: Relative Gibbs free energy diagrams for the mechanism with H_2.

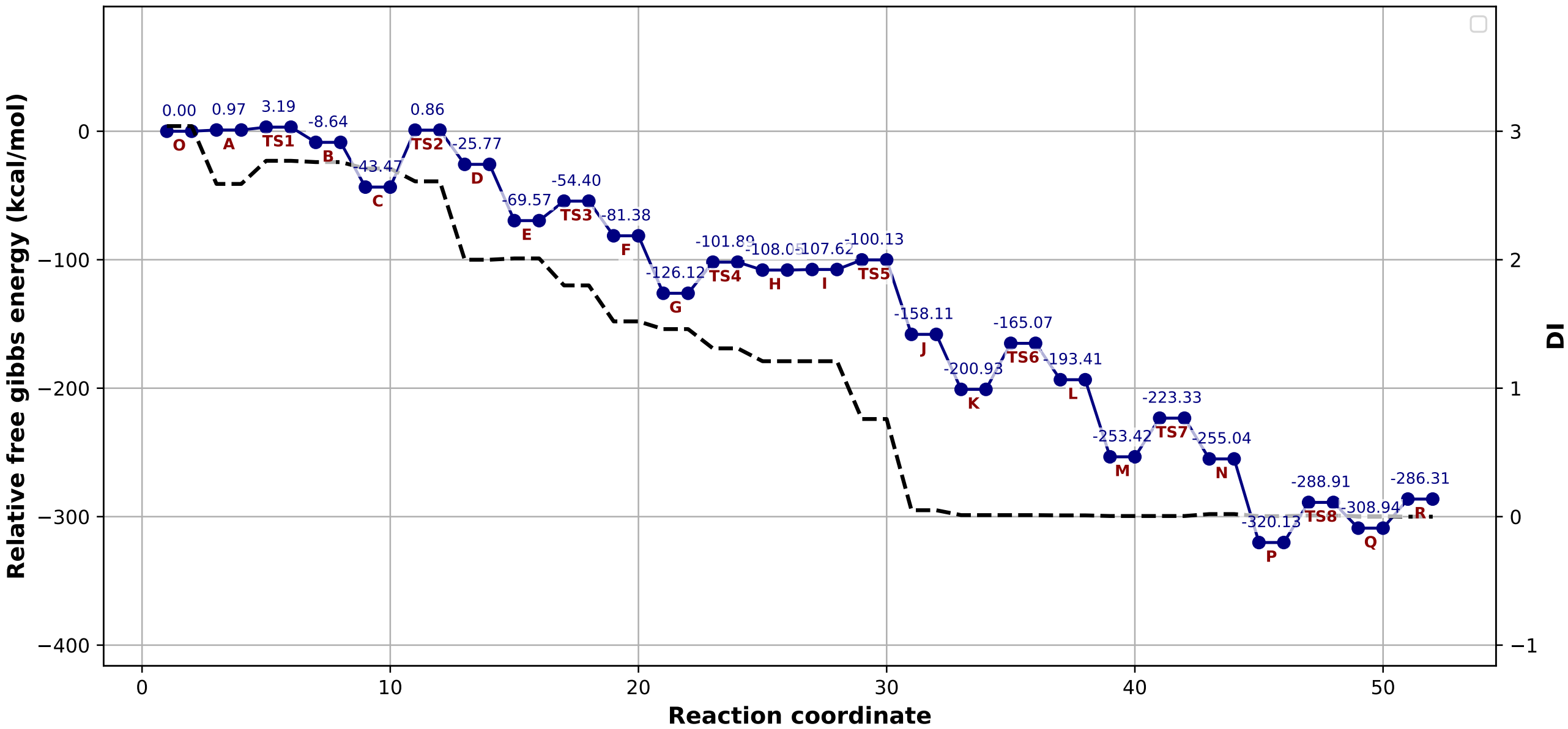

Figure S7: Relative Gibbs free energy diagrams for the mechanism A with $MoSn_3$ as catalyst (continuous line) alongside the evolution of the DI of the N−N bond through the reaction mechanism (dashed line).

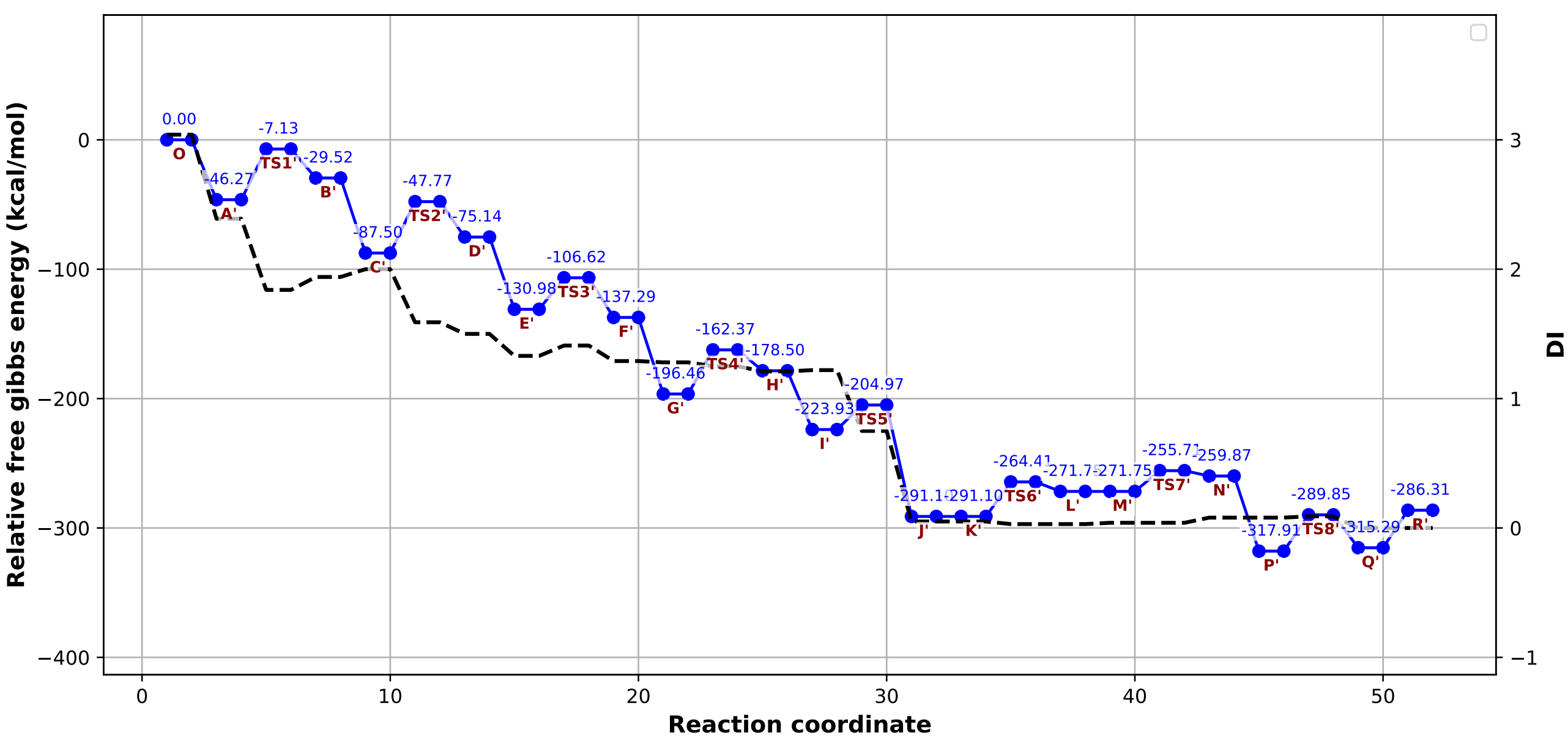

Figure S8: Relative Gibbs free energy diagrams for the mechanism B with $MoSn_3$ as catalyst (continuous line) alongside the evolution of the DI of the N−N bond through the reaction mechanism (dashed line).

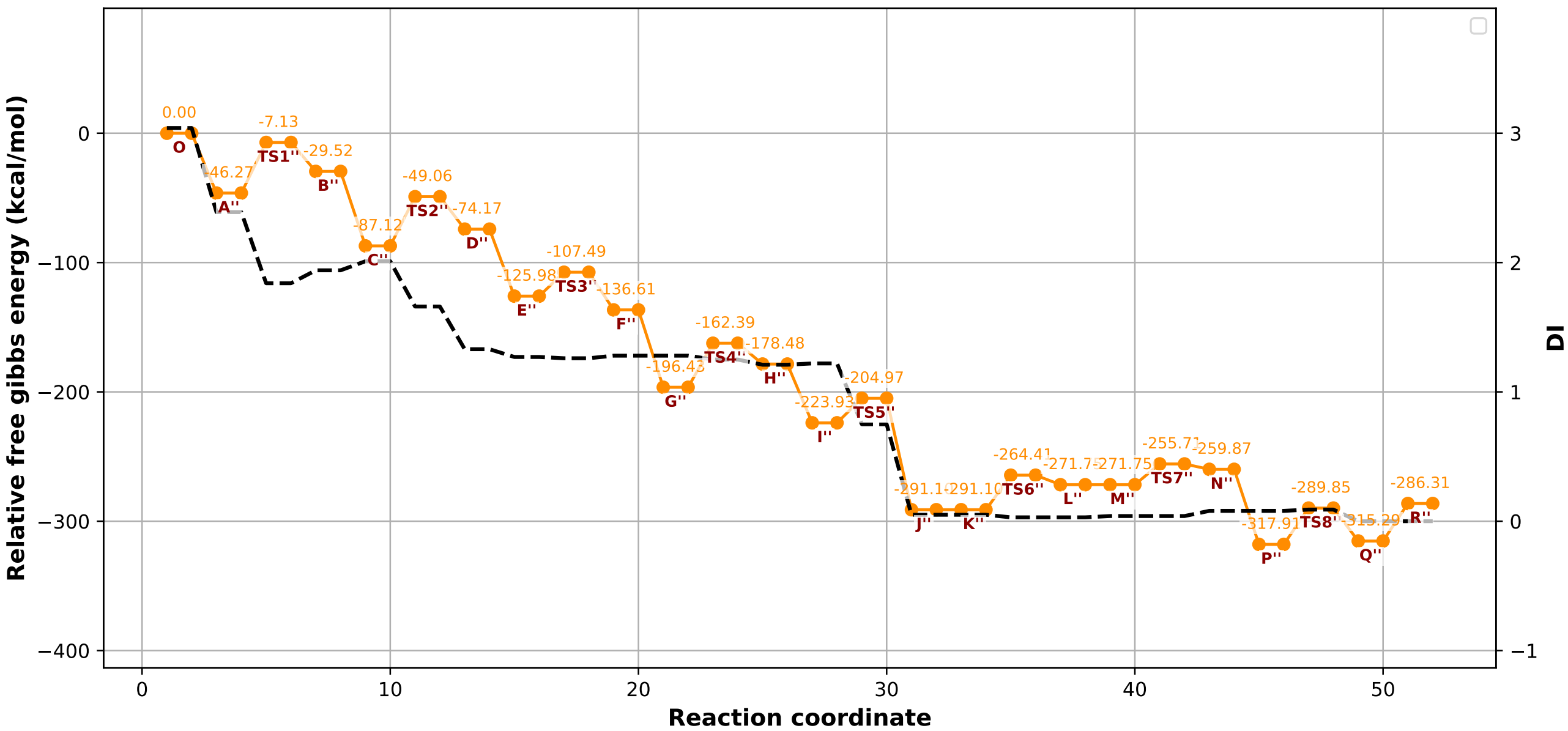

Figure S9: Relative Gibbs free energy diagrams for the mechanism C with $MoSn_3$ as catalyst (continuous line) alongside the evolution of the DI of the N−N bond through the reaction mechanism (dashed line).

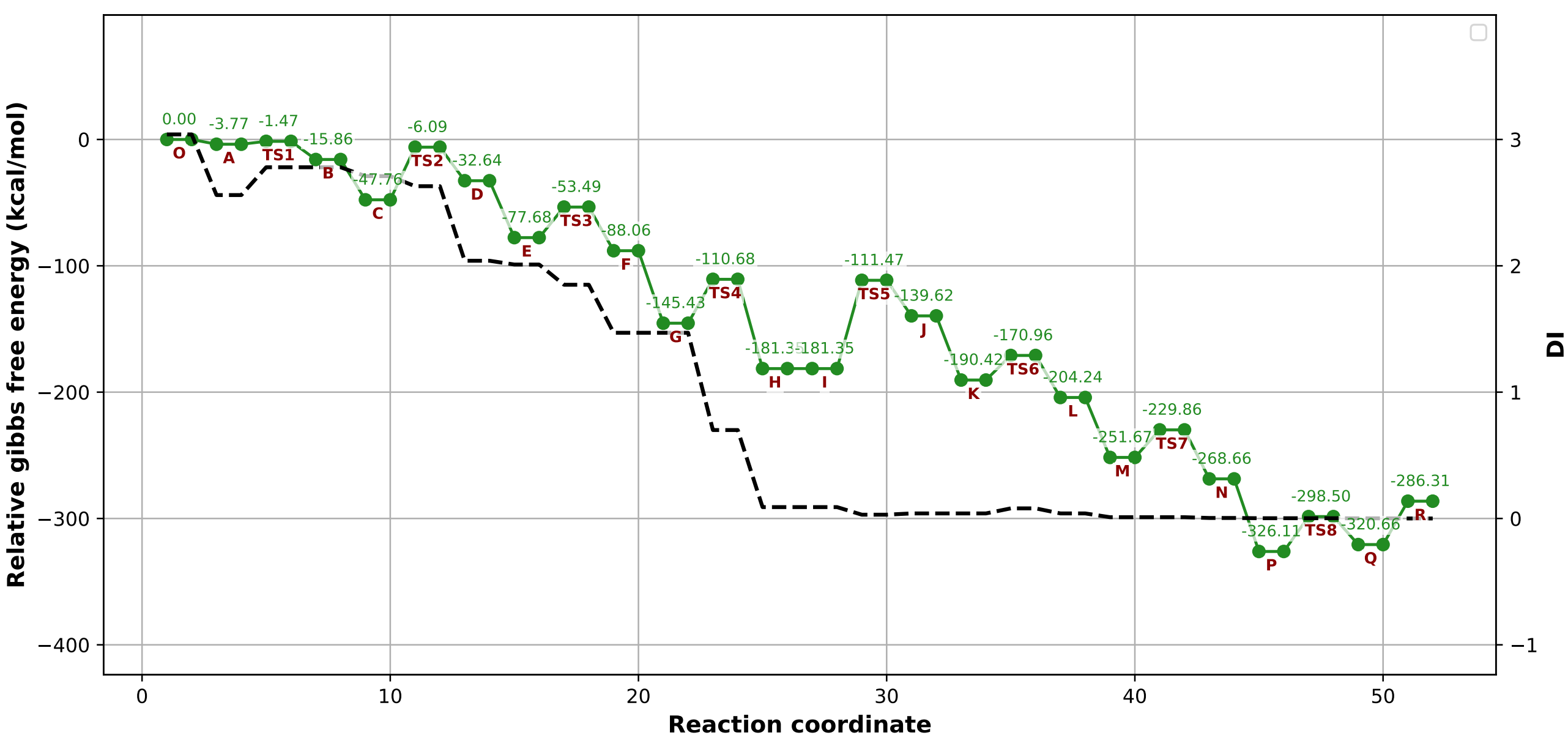

Figure S10: Relative Gibbs free energy diagrams for the mechanism A with $MoSn_5$ as catalyst (continuous line) alongside the evolution of the DI of the N−N bond through the reaction mechanism (dashed line).

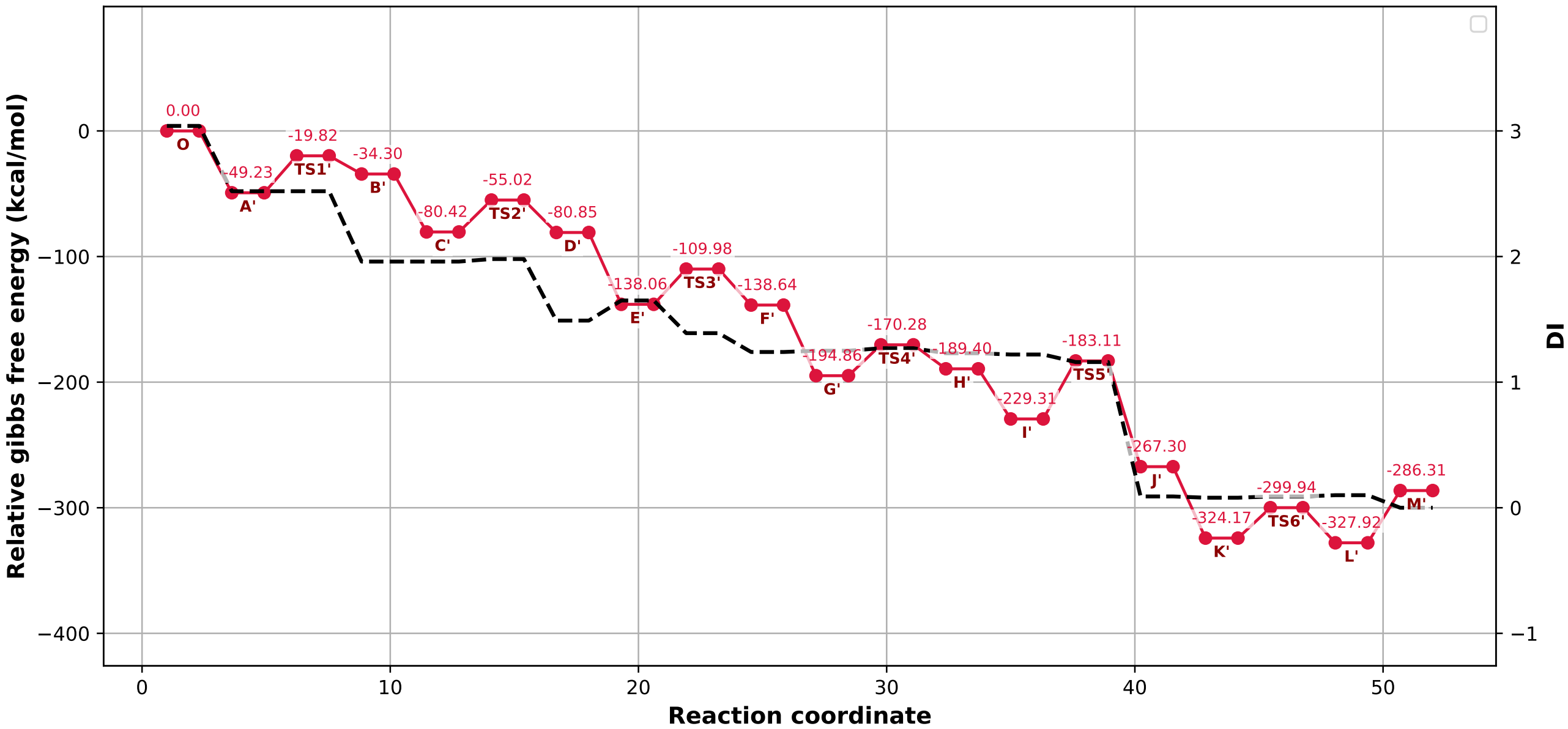

Figure S11: Relative Gibbs free energy diagrams for the mechanism B with $MoSn_5$ as catalyst (continuous line) alongside the evolution of the DI of the N−N bond through the reaction mechanism (dashed line).

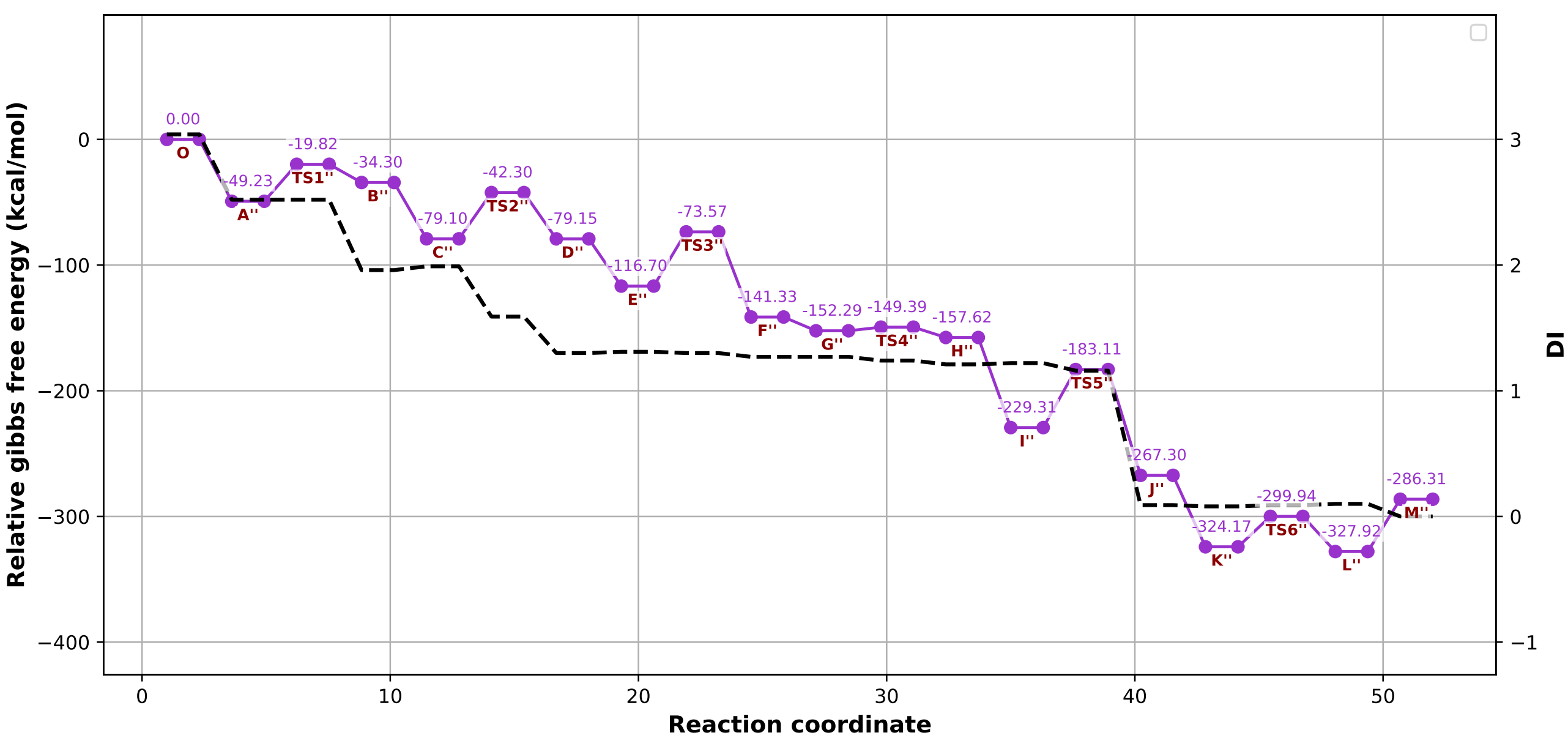

Figure S12: Relative Gibbs free energy diagrams for the mechanism C with $MoSn_5$ as catalyst (continuous line) alongside the evolution of the DI of the N−N bond through the reaction mechanism (dashed line).

Wave function analyses

Table S1: QTAIM charge (Q_H) and ΔQ_H values for the H atoms adsorbed onto the $MoSn_3$ and $MoSn_5$ clusters for every mechanism explored.

Reactant	Q_H	TS	Q_H	ΔQ_H
$MoSn_3$ A **C**	-0.34	$MoSn_3$ A **TS2**	0.04	-0.38
$MoSn_3$ A **E**	-0.31	$MoSn_3$ A **TS3**	-0.09	-0.23
$MoSn_3$ A **G**	-0.33	$MoSn_3$ A **TS4**	0.03	-0.35
$MoSn_3$ A **K**	-0.43	$MoSn_3$ A **TS6**	-0.03	-0.39
$MoSn_3$ A **M**	-0.36	$MoSn_3$ A **TS7**	0.02	-0.38
$MoSn_3$ A **P**	-0.40	$MoSn_3$ A **TS8**	0.01	-0.41
$MoSn_3$ B **A'**	-0.45	$MoSn_3$ B **TS1'**	0.35	-0.80
$MoSn_3$ B **C'**	-0.41	$MoSn_3$ B **TS2'**	0.01	-0.42
$MoSn_3$ B **E'**	-0.41	$MoSn_3$ B **TS3'**	-0.03	-0.38
$MoSn_3$ B **G'**	-0.45	$MoSn_3$ B **TS4'**	0.02	-0.47
$MoSn_3$ B **I'**	-0.41	$MoSn_3$ B **TS5'**	0.01	-0.42
$MoSn_3$ B **P'**	-0.45	$MoSn_3$ B **TS8'**	-0.03	-0.42
$MoSn_3$ C **A"**	-0.45	$MoSn_3$ C **TS1"**	0.35	-0.80
$MoSn_3$ C **C"**	-0.41	$MoSn_3$ C **TS2"**	-0.04	-0.37
$MoSn_3$ C **E"**	-0.39	$MoSn_3$ C **TS3"**	-0.04	-0.35
$MoSn_3$ C **G"**	-0.45	$MoSn_3$ C **TS4"**	0.02	-0.47
$MoSn_3$ C **I"**	-0.41	$MoSn_3$ C **TS5"**	0.01	-0.42
$MoSn_3$ C **P"**	-0.45	$MoSn_3$ C **TS8"**	-0.03	-0.42
$MoSn_5$ A **C**	-0.29	$MoSn_5$ A **TS2**	0.05	-0.34
$MoSn_5$ A **E**	-0.29	$MoSn_5$ A **TS3**	-0.03	-0.26
$MoSn_5$ A **I**	-0.40	$MoSn_5$ A **TS5**	-0.05	-0.35
$MoSn_5$ A **K**	-0.38	$MoSn_5$ A **TS6**	-0.09	-0.29
$MoSn_5$ A **M**	-0.39	$MoSn_5$ A **TS7**	-0.01	-0.38
$MoSn_5$ A **P**	-0.42	$MoSn_5$ A **TS8**	0.01	-0.43
$MoSn_5$ B **A'**	-0.37	$MoSn_5$ B **TS1'**	-0.01	-0.36
$MoSn_5$ B **C'**	-0.29	$MoSn_5$ B **TS2'**	0.02	-0.31
$MoSn_5$ B **E'**	-0.40	$MoSn_5$ B **TS3'**	0.02	-0.41
$MoSn_5$ B **G'**	-0.41	$MoSn_5$ B **TS4'**	-0.02	-0.39
$MoSn_5$ B **I'**	-0.39	$MoSn_5$ B **TS5'**	0.34	-0.73
$MoSn_5$ B **K'**	-0.41	$MoSn_5$ B **TS6'**	-0.02	-0.39
$MoSn_5$ C **A"**	-0.37	$MoSn_5$ C **TS1"**	-0.01	-0.36
$MoSn_5$ C **C"**	-0.31	$MoSn_5$ C **TS2"**	0.10	-0.40
$MoSn_5$ C **E"**	-0.30	$MoSn_5$ C **TS3"**	-0.01	-0.30
$MoSn_5$ C **G"**	-0.33	$MoSn_5$ C **TS4"**	0.19	-0.52
$MoSn_5$ C **I"**	-0.39	$MoSn_5$ C **TS5"**	0.34	-0.73
$MoSn_5$ C **K"**	-0.41	$MoSn_5$ C **TS6"**	-0.02	-0.39

XYZ files

In this link

`https://doi.org/10.5281/zenodo.20585190`

the interested reader can download the XYZ files corresponding to the intermediaries and transition states addressed in this investigation. The corresponding geometries were computed with the PBE/def2-SVP approximation.
